# Rhizobial Symbiosis Enhances Salinity Tolerance in Legumes Through Coordinated Physiological and Molecular Responses: Evidence From Common Vetch and Pea

**DOI:** 10.1111/pce.70790

**Published:** 2026-08-04

**Authors:** María Isabel López‐Román, Lucía Zurita, Cristina Castaño‐Herrero, Lucía De la Rosa, Elena Ramírez‐Parra

**Affiliations:** ^1^ Centro de Biotecnología y Genómica de Plantas, (CBGP, UPM‐INIA/CSIC) Instituto Nacional de Investigación y Tecnología Agraria y Alimentaria, Consejo Superior de Investigaciones Científicas Universidad Politécnica de Madrid. Campus de Montegancedo Pozuelo de Alarcón Spain; ^2^ Centro de Recursos Fitogenéticos, (CRF‐INIA/CSIC), Instituto Nacional de Investigación y Tecnología Agraria y Alimentaria Consejo Superior de Investigaciones Científicas Alcalá de Henares Spain

**Keywords:** common vetch, pea, Rhizobium, salinity stress, symbiosis, transcriptomic response

## Abstract

Soil salinisation severely constrains crop production. Legumes rely on rhizobial symbiosis for sustainable nitrogen acquisition. Although salinity is known to impair nodulation and nitrogen fixation, how symbiosis influences plant tolerance to salt stress remains unclear. In this study, we integrate physiological, biochemical, transcriptomic, ionomic, and metabolomic analyses to investigate the role of rhizobial symbiosis in salinity tolerance in *Vicia sativa* (common vetch) and *Pisum sativum* (pea). In both species, nodulated plants exhibited markedly enhanced survival and grain production under prolonged salt stress compared with nitrogen‐fertilised (N‐fed) controls. Under moderate salinity, nodulation helped maintain water status and stomatal conductance, reduced Na^+^ accumulation while improving K^+^ retention, and attenuated osmotic and oxidative stress, as evidenced by lower proline and malondialdehyde levels. Transcriptomic profiles in vetch revealed that symbiosis is associated with changes in the plant responses toward growth and microbial signalling, reducing the strong activation of stress pathways that typically occurs in non‐nodulated plants. Ionomic and metabolomic data further showed that nodulated plants preserve nutrient balance and maintain a more stable carbon‐nitrogen metabolism under salinity. These findings highlight the potential of Rhizobium inoculation to enhance crop resilience in salt‐affected agroecosystems and demonstrate that symbiosis correlates with a more efficient and physiologically moderated acclimation to salinity in legumes.

## Summary statement

Rhizobial symbiosis enhances salinity tolerance in *Vicia sativa* and *Pisum sativum* by improving water status, maintaining a favourable ion balance, reducing osmotic and oxidative stress, and moderating transcriptomic, ionomic and metabolomic responses under salt stress.

## 1 Introduction

Salinity is a major constraint for crop productivity worldwide. Recent FAO reports highlight soil salinisation as a growing threat, driven by climate change, severe drought, and anthropogenic activities such as unsustainable irrigation management and overexploitation of natural resources, especially in arid and semi‐arid regions (FAO 2024). Salt stress reduces grain and biomass production, inhibits seed germination and cell elongation, and restricts overall plant growth. As a consequence of these effects, plants develop different strategies to adapt and survive under salinity, including three main response mechanisms: osmotic adjustment, redox regulation, and ionic homeostasis (Zhao et al. 2021; Xiao and Zhou 2022; Fu and Yang 2023). The osmotic response involves the regulation of water status and stomatal conductance, balancing transpiration with CO_2_ assimilation, a trade‐off that may compromise photosynthesis. High salinity promotes the synthesis of osmolytes, such as proline, which help prevent cellular dehydration and also function as antioxidants by scavenging reactive oxygen species (ROS) (Ben Rejeb et al. 2014; van Zelm et al. 2020; Jogawat 2019; Hare et al. 1998; Bartels and Sunkar 2005). ROS accumulation is controlled by enzymatic and non‐enzymatic antioxidant systems that prevent oxidative damage (Li et al. 2019; Kesawat et al. 2023); (Racchi 2013; Khatun et al. 2021; Khazaei and Aghaz 2017). Salinity leads to the accumulation of Na^+^ and Cl^−^, disrupting Na^+^/K^+^ homeostasis, which is essential for cellular function. Plants manage this imbalance through ion transport systems that mediate Na^+^ exclusion and K^+^ retention (Zhao et al. 2021; Munns and Tester 2008; Balasubramaniam et al. 2023; Ali et al. 2023).

These complex and integrative mechanisms act at different levels in both the root and shoot. Thus, at the shoot level, salt promotes stomatal closure, decreases water content, inhibition of photosynthesis and leaf chlorosis and senescence. At the root level, salt stress induces an inhibition of water and nutrient uptake and reduction of root growth (Nadeem et al. 2019). Together, these mechanisms enable plants to adapt to high salinity by maintaining water balance, limiting oxidative stress, and regulating ion concentrations. Phytohormones contribute to plant responses to salt stress, with abscisic acid (ABA) and brassinosteroids (BR) among the pathways associated with the regulation of stomatal behaviour, ion balance, and stress acclimation under saline conditions (Xiao and Zhou 2022; Bharath et al. 2021; Guo et al. 2024; Vikram et al. 2022; Wang et al. 2020).

Members of the family *Fabaceae* are not only nutritious but also cost‐effective food sources, playing a crucial role in providing more than 33% of dietary human and animal protein intake (Smýkal et al. 2015). In addition, legumes are essential for sustainable agriculture due to their capability to form symbiotic relationships with nitrogen‐fixing bacteria, which improves soil fertility and reduces reliance on synthetic fertilisers, contributing to sustainable agriculture (Soumare et al. 2020). Legumes are highly sensitive to salinity stress, particularly at the seedling and developmental stages (Sehrawat et al. 2015; Nadeem et al. 2019; Bouzroud et al. 2023).

Severe salinity deleteriously affects the establishment and functioning of rhizobium‐legume symbiosis, disrupting nodulation and nitrogen fixation (Zahran 1999). On the other hand, recent studies show that the legumes that establish symbiosis with rhizobia are more resistant to abiotic stresses. According to some research rhizobial symbiosis in several legume species induce drought tolerance (Antolín et al. 1995; Álvarez‐Aragón et al. 2023; Amine‐Khodja et al. 2022; Frechilla et al. 2000; Lodeiro et al. 2000; Staudinger et al. 2016), but only to a lesser extent in a less characterised way, to salinity (Turetschek et al. 2019; Wekesa et al. 2022).

While the impact of salinity on rhizobial symbiosis has been widely studied, how symbiosis influences plant responses to salinity remains poorly understood. Within legume diversity, *Vicia sativa* (common vetch) and *Pisum sativum* (pea) represent two agronomically relevant species in temperate and Mediterranean agrosystems increasingly affected by soil salinisation. Both crops contribute substantially to nitrogen inputs through symbiosis and exhibit well‑documented sensitivity to salt stress, while also differing in root architecture and nodulation traits. These characteristics make them suitable models to investigate how rhizobial symbiosis modulates plant responses to salinity (Ramírez‐Parra and De la Rosa 2023; Khan et al. 2022).

The main objective of this work is to analyse the impact of rhizobial symbiosis on salt stress tolerance in *Vicia sativa* and *Pisum sativum* by analysing physiological, biochemical, and molecular responses in nodulated plants and non‐inoculated but nitrogen‐supplemented controls. Integrating these data will provide insights into the mechanisms underlying the mitigation of salinity effects in nodulated plants.

## 2 Materials and Methods

### 2.1 Biological Materials and Growth Conditions

We utilised common vetch (*V. sativa* cultivar Senda) and pea (*P. sativum* cultivar Frisson) for the plant assays. Seeds were surface sterilised with a solution of 96% ethanol and 12% bleach, then germinated on 1% agar plates. Inoculation experiments were conducted in sterile Leonard jars filled with a mixture of sand and vermiculite (1:1 ratio) as the growing substrate and a nitrogen‐free hydroponic nutrient solution (Brito et al. 1994). The seedlings were inoculated with 1 mL of rhizobial culture in the early stationary phase. Our inoculant was *Rhizobium leguminosarum* bv. *viciae* (Rlv) strain V31, previously isolated from soil in our laboratory (Álvarez‐Aragón et al. 2023). Bacterial growth‐curve kinetics were determined by growing *Rlv* strain V31 at 28°C in Universal Minimal Salt (UMS) medium (Rubia et al. 2020) at different salt concentrations (0, 100, 200, 400 and 600 mM NaCl) and by optical density‐based growth determination using a Bioscreen C Pro (Thermo Fisher Scientific). For plants receiving nitrogen (N‐fed), we supplemented the nutrient solution with 10 mM NH_4_NO_3,_ 10 days after sowing. Vetch and pea plants were cultivated in a greenhouse under a 16‐h light/8‐h dark photoperiod at 22 ± 1°C. Leaf samples were collected from pea and vetch plants at 39 days old, unless otherwise specified. To control for the effect of nitrogen availability on symbiosis, additional conditions were established under different nitrogen regimes. Plants were grown under absent nitrogen conditions, allowing active nodulation, as well as under high nitrogen supply (10 mM NH_4_NO_3_). Under high nitrogen conditions, nodulation and nitrogenase activity were suppressed, and plants displayed a non‐symbiotic phenotype. In parallel, non‐inoculated plants grown under low nitrogen conditions were included as controls to assess nitrogen deficiency.

For moderate‐salinity treatments, we placed 4‐week‐old plants in Leonard jars with hydroponic nutrient solution supplemented with 150 mM NaCl for 11 additional days. For survival assays, prolonged salinity treatments were applied to plants initially grown for 4 weeks. After that time, plants were treated with 150 mM NaCl prolonged for 3 additional weeks until clear symptoms of severe salt damage appeared and then rewatered with hydroponic nutrient solution (without NaCl) again for 2 weeks. The experiment timeline of both treatments is shown in Supporting Information S2: Figure S1. Each assay was replicated three times with a minimum of 12 plants per experiment in a randomised design.

### 2.2 Measurement of Stress‐Associated Parameters: Stomatal Conductance, Relative Water Content (RWC), Pigment and Osmolyte Determinations, and Lipid Peroxidation

Stomatal conductance was assessed in duplicate on fully expanded mature leaves from a total of eight plants per treatment as indicated, utilising a steady‐state leaf porometer (SC‐1, Decagon Devices, LabFerrer, Spain), as described by Álvarez‐Aragón et al. (2023). For the estimation of RWC, the shoot of 4‐week‐old plants exposed to salt stress for 11 days was detached and immediately weighed (FW, fresh weight). Samples were immediately hydrated for 16 h in deionised water to full turgidity and weighed (TW). To estimate the dry weight (DW), shoot samples were oven‐dried (65°C) for 5 days. The percentage of plant RWC was calculated as 100 × (FW − DW)/(TW − DW).

For pigment and osmolyte determination, we utilised leaves from 4‐week‐old plants grown in the greenhouse and subjected them to both control and salt treatments for 11 days. Chlorophyll content was determined spectrophotometrically after acetone extraction as described (Arnon 1949). Lipid peroxidation was measured by determining the malondialdehyde (MDA) content in mature leaves using the thiobarbituric acid (TBA) reaction (Heath and Packer 1968). Proline levels were determined as indicated (Bates et al. 1973). Anthocyanins were extracted using the acidified methanol method previously described (Wade et al. 2003). Statistical analysis of these physiological and biochemical data involved at least three independent experiments, each with a minimum of six plants per experiment.

### 2.3 Nodulation, Symbiotic Activity and Nitrogen and Carbon Content

Nodules from 39‐day‐old plants (with or without moderate salt treatments) were collected and promptly processed for the measurement of their fresh weight and quantification of their number and activity. Nitrogen fixation activity of nodules was estimated by the acetylene reduction test as previously described (Ruiz‐Argüeso et al. 1978) using a Shimadzu GC‐8 device. The shoots of the plants were subjected to oven‐drying at 65°C for 1 week, tissues were weighed, ground, and the total nitrogen and carbon content were determined using the Dumas Method with a TruMac C/N analyser (Leco). Similarly, the elemental analysis of minerals, including K/Na levels, was estimated by ICP‐OES (Inductively Coupled Plasma‐Optical Emission Spectrometry) by Thermo iCAP 6500 Duo ICP‐AES Spectrometer (Thermo Scientific) at the Ionomics Platform of CEBAS (CSIC, Spain). Statistical analysis was conducted based on at least three independent experiments, each involving seven plants per experiment.

### 2.4 Transcriptome Analysis: RNA Extraction and Library Construction From *V. Sativa* Samples

Shoot and root samples of 39‐day‐old vetches and peas (28‐day‐old plants plus 11 days of salt treatment) from the four different treatments (nodulated control, N‐fed control, nodulated/salt stress and N‐fed/salt stress) were collected and frozen in liquid nitrogen in pools of four plants per pool and three pools per treatment. Total RNA was extracted using the Plant RNA Extraction Kit (Omega). The extracted RNA quantity and quality were determined using an Invitrogen Qubit 3.0 Fluorometer (Thermo Fisher Scientific, USA) and Agilent Bioanalyzer 5400 (RIN > 7.5). For vetch cDNA libraries (12 from root and 12 from shoot samples) construction, the mRNA was purified from total RNA (5 μg) using poly‐T oligo‐attached magnetic beads. After fragmentation, the first‐strand cDNA was synthesised using random hexamer primers. The libraries were checked with Qubit and real‐time PCR for quantification and a bioanalyzer for size distribution detection and sequenced according to Illumina's recommendations. The total pools of each of the 24 libraries were sequenced by paired‐end sequencing (151 × 2) using Illumina Novogene Facilities (Germany). Genome‐wide transcriptomic analyses (including DEG identification, GO enrichment, and transcription factor [TF] profiling) were performed exclusively in Vicia sativa. In Pisum sativum, molecular analyses were limited to targeted expression analysis of selected stress‐responsive genes by RT‐qPCR.

### 2.5 Data Analysis for Transcriptome Sequencing: De Novo Assembly and Functional Annotation

The quality of the raw read data in FASTQ format was first processed through in‐house scripts. A total of 647 million raw reads (633 million clean or trimmed reads) were obtained by trimming reads containing adapters, removing poly‐N sequences and reads with low quality from raw data. At the same time, Q20, Q30 and GC content of the clean data were calculated. Detailed procedures for RNAseq processing are included in Supporting Information S1: Table S1.

The de novo assembly and functional annotation of vetch sequences were performed using Trinity for reconstruction of transcriptomes from RNA‐seq data. After that, CORSET software was used to remove the redundancy of Trinity results and hierarchical clustering was performed based on multiple mapping events and expression patterns. Finally, BUSCO was employed to quantitatively assess the completeness of the transcriptome by evaluating the presence of expected gene content. After quality control, trimming, data clean‐up process, and de novo assembly, the resulting unigene sets were merged into 65 129 high‐quality and non‐redundant unique unigenes, with a length between 301 and 17 171 nucleotides, an average length of 1311 nucleotides and an N50 length of 2097 nucleotides (Summarised data in Supporting Information S1: Table S2). For gene functional annotation, seven databases were applied, including NCBI Nucleotide BLAST (NT); NCBI non‐redundant Protein (NR); SwissProt; and Eukaryotic Orthologous Groups of proteins (KOG). DIAMOND e‐value cut‐off of 1.0E‐5; top10 alignment results are shown for these four databases. Kyoto Encyclopedia of Genes and Genomes (KEGG; threshold 0.01); Protein family (Pfam; threshold 1E‐6) and Gene Ontology (GO; threshold 1E‐6) were also blasted. The success rate of annotation of unigenes in each database is indicated in Supporting Information S1: Table S3. In total, 48246 (78.4%) transcripts were successfully annotated in at least one database. GO classification was applied (Supporting Information S2: Figure S2A). Briefly, the major biological processes (BP) category was overrepresented by cellular process (31.1%), metabolic process (26.2%), biological regulation (10.1%) and regulation (9.7%). The notable molecular functions (MFs) subgroups were binding (44%), catalytic activity (32.3%), transporter activity (6.5%), and ATP‐dependent (4.6%). In the cellular components (CCs) group, cellular anatomical entity (66.8%) and protein‐containing complex (30.2%) were the largest GO subgroups. A total of 12 060 transcripts were classified into the 24 EggNOG pathways (Supporting Information S2: Figure S2B). The most overrepresented categories were posttranslational modification, protein turnover and chaperones (12.3%), translation, ribosomal structure and biogenesis (10.9%), general function prediction (10.5%), signal transduction (7.4%) and intracellular trafficking, secretion, and vesicular transport (6.8%).

### 2.6 Differential Expressed Gene Analysis and Functional Enrichment

For the analysis of gene expression levels, clean read data was mapped back to the assembled transcriptome from Bowtie, and quantification was estimated by the RSEM algorithm, converting them into FPKM (Fragments Per Kilobase of transcript sequence per Million base pairs sequenced) values. Similarity between different biological replicates and different treatments was corroborated through correlation analysis using Pearson's coefficient of the normalised value. The analysis of differential expression between two treatments (three biological replicates per condition) was performed using DESeq. 2 R (Stacklies et al. 2007) using a model based on the negative binomial distribution for determination of statistical significance. Significant differential expression analysis of two conditions was performed using the edgeR R package with a threshold *p* < 0.05 (corrected by FDR estimation Benjamini and Hochberg methods) and |log_2_(Fold Change)|> 1.

For the statistical GO and KEGG‐pathways enrichment analysis of each set of DEGs, the clusterProfiler R package and KOBAS software were used (*p* < 0.05), respectively. Hierarchical clustering of the DEGs was performed using complete linkage and Euclidean distance metrics, and the results were visualised with the Morpheus online tool (https://software.broadinstitute.org/morpheus/). Scientific illustrations were generated using BioRender software (BioRender.com).

### 2.7 Real‐Time Quantitative PCR Analysis

RNA samples were extracted following the protocol outlined for transcriptomic analysis and then utilised for cDNA synthesis. Retrotranscription was performed using the NZY First‐Strand cDNA Synthesis Kit with oligodT primers (NZYtech, Portugal). Real‐time quantitative RT‐PCR was carried out on the LightCycler480 II Roche system using the LightCycler 480 SYBR Green I (Roche). Transcript levels of Glyceraldehyde‐3‐Phosphate‐Dehydrogenase (VsGADPDH and PsGADPDH) were utilised for normalisation/standardisation purposes. Data obtained from two independent experiments were conducted in triplicate. Sequence primers for vetch (Vs) and pea (Ps) quantitative PCR (qPCR) are detailed in Supporting Information S1: Table S4.

### 2.8 Metabolomic and Ionomic Analyses

The quantification of primary metabolites (amino acids, sugars and organic acids) was performed using ^1^H‐NMR at the Metabolomics Platform of CEBAS (CSIC, Spain). The elemental‐ionomic analyses of minerals were estimated by inductively coupled plasma‐optical emission spectrometry (ICP‐OES) using a Thermo iCAP 6500 Duo ICP‐AES Spectrometer (Thermo Scientific) at the Ionomics Platform of CEBAS (CSIC, Spain).

### 2.9 Statistical Analysis

The physiological and biochemical data were subjected to statistical analysis using the Excel add‐in Real Statistic pack. Student's *t*‐test was employed for comparing two groups. Comparisons between the control and different treatments were conducted using the one‐way ANOVA test (*p* < 0.05). Means were compared using Tukey's test, and different letters indicate significant differences, assigned in descending order, corresponding to the highest mean. Principal Component Analysis (PCA) and multivariable correlation analysis were conducted using the Statgraphics Centurion v18.1 software.

## 3 Results

### 3.1 Symbiosis Promotes Survival Under Extreme Salinity Conditions

To investigate whether rhizobial symbiosis enhances plant tolerance to prolonged salinity conditions, we initially characterised the salt tolerance of the rhizobial strain, Rlv V31, used in this study. This strain tolerated high NaCl levels, showing only marginal growth reduction between 100 and 200 mM (Figure 1A), which justified selecting 150 mM NaCl as the treatment concentration. Using this condition, 4‑week‑old nodulated vetch plants and N‑fed controls were exposed to salt stress for 3 weeks before undergoing an 8‑day recovery phase (Figure 2). Under these severe conditions, nodulated plants displayed markedly higher survival than N‑fed plants. Specifically, 70.8% of nodulated vetch plants survived, whereas survival in N‑fed plants dropped to 4.2%, representing a ~17‑fold increase in survival associated with symbiosis (4.2% ± 7.2 vs. 70.8% ± 14.4; Figure 2A,B). Moreover, nodulated plants produced ~28‑fold higher grain yield than N‑fed plants under the same stress conditions (0.04 ± 0.02 vs. 0.99 ± 0.20 g per plant; Figure 2C).

**Figure 1:**
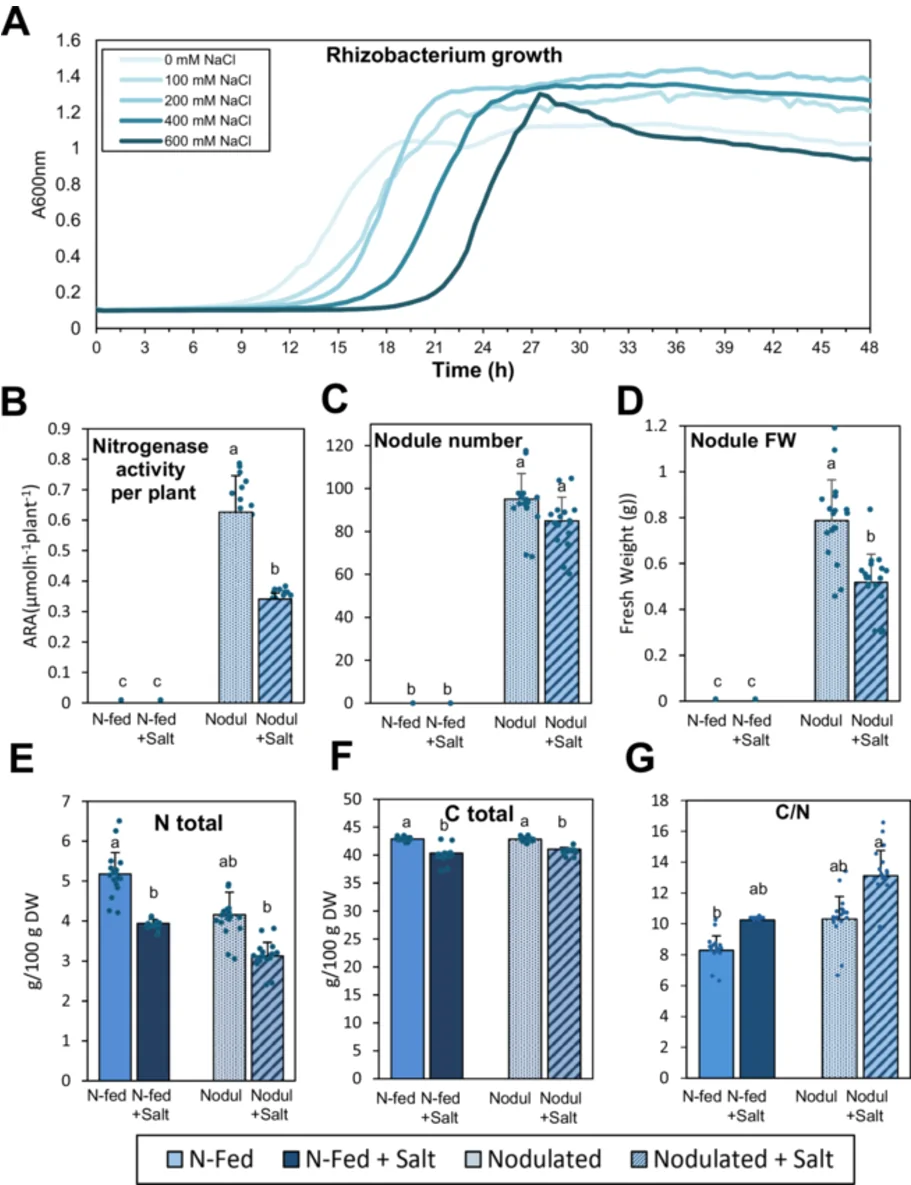
Effect of salt on Rhizobium‐vetch symbiosis. (A) Growth curve of *Rhizobium leguminosarum* strain V31 in USM medium with different NaCl concentrations. (B) Nitrogenase activity per plant by Acetylene Reduction Assay (ARA) method. (C) Nodule number per plant. (D) Nodule total fresh weight per plant. Quantification of N (E) and C (F) content and C/N ratios (G) on shoot at indicated conditions. All measurements in panels B–G were performed on 39‐day‐old plants under moderate salinity (150 mM NaCl; 11‐day treatment applied to 4‐week‐old plants). At least three independent experiments were performed (*n* = 6 plants each). Values are means ± SD. Letters indicate significant differences. *p* < 0.01 (ANOVA and Tukey HSD test).

**Figure 2:**
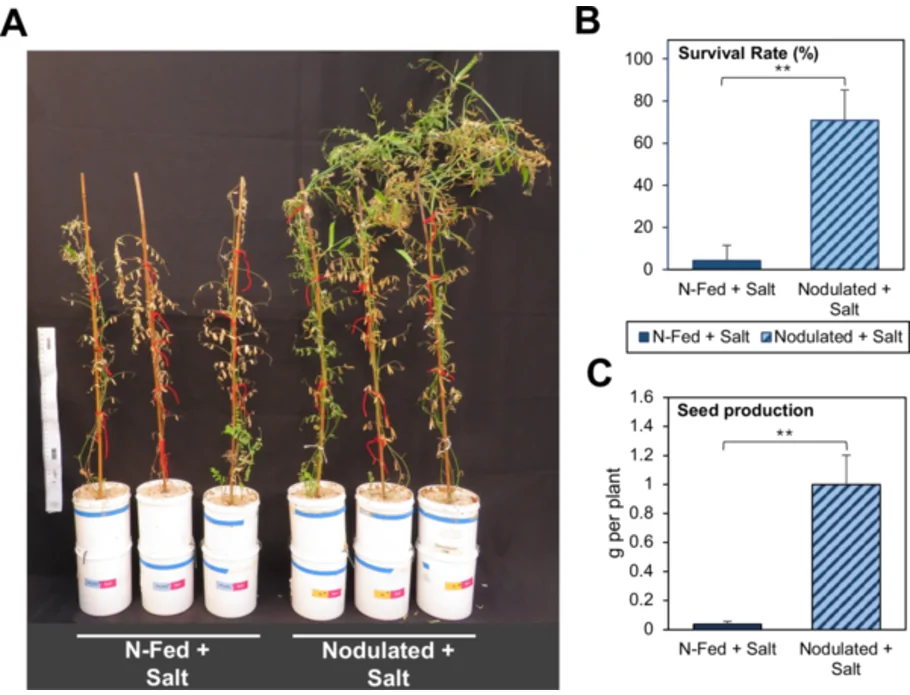
Enhanced salt tolerance in nodulated vetch plants relative to nitrogen‐fertilised plants. (A) Four‐week‐old nodulated and N‐fed plants were exposed to salt stress (150 mM NaCl) for 3 weeks, and then the stress was removed for 8 days. (B) Survival rate (B) and seed production (C) yield in nodulated and N‐fed plants after 3 weeks of salt stress and 23 days of recovery. Student *t*‐test (**) *p* < 0.005. [Color figure can be viewed at wileyonlinelibrary.com]

A similar trend was observed in pea plants (Supporting Information S2: Figure S3). Nodulated pea plants showed a ~11‑fold increase compared with N‑fed plants. Seed production was also dramatically higher (~107‑fold increase) in nodulated plants versus N‑fed plants.

### 3.2 Effects of Salinity on Legume Symbiosis

Although, as mentioned above, Rhizobium strain Rlv V31 exhibits high salt tolerance (Figure 1A), extreme salinity still has a deleterious effect on the symbiosis (Supporting Information S2: Figure S10), leading to strong inhibition of nitrogenase activity and nodule development in vetch plants. In contrast, under moderate salinity (150 mM NaCl; 11‐day treatment applied over 4‐week plants), we observed only a partial reduction in nitrogenase activity compared with untreated nodulated control plants (Figure 1B). Salinity did not significantly affect nodule number (Figure 1C), but it reduced nodule fresh weight (Figure 1D). A similar pattern was observed in pea, where moderate salinity also reduced nitrogenase activity and nodule biomass without affecting nodule number (Supporting Information S2: Figure S4A–C).

Although nitrogenase activity was significantly reduced under salt stress, it remained above 50% of control values. Total nitrogen content showed a moderate decrease under these conditions and remained close to that of N‐fed plants, both in vetch and in pea (Figure 1E; Supporting Information S2: Figure S4D). Total carbon content was only slightly affected (Figure 1F; Supporting Information S2: Figure S4E,F). As a consequence, the C/N ratio displayed a moderate increase under salt stress, particularly in nodulated plants (Figure 1G; Supporting Information S2: Figure S4E,F).

To control for the effect of nitrogen availability on symbiosis, additional conditions were established under different nitrogen regimes. Plants were grown in nitrogen‐deficient media, allowing active nodulation, as well as under high nitrogen supply (10 mM NH_4_NO_3_). Under high nitrogen conditions, nodulation and nitrogenase activity were suppressed, and plants displayed a non‐symbiotic phenotype. In parallel, non‐inoculated plants grown under low nitrogen conditions were included as controls to assess nitrogen deficiency. These control experiments are shown in Supporting Information S2: Figure S11.

### 3.3 Physiological and Biochemical Characterisation of Salt‐Tolerance

To evaluate how rhizobial symbiosis modulates the physiological response of common vetch to salinity, we analysed RWC in nodulated and nitrogen‑fertilised plants exposed to salt stress. In our assays, salt stress caused a reduction in RWC in both treatments; however, the magnitude of this decrease differed markedly. N‑fed plants showed a 36% reduction in RWC, whereas nodulated plants exhibited only an 8% decrease (Figure 3A). Because water loss is closely linked to stomatal behaviour, we next examined stomatal conductance. Consistent with the RWC data, salinity induced a stronger decline in stomatal conductance in non‐nodulated N‑fed plants (61% reduction) compared with nodulated plants (only 39% reduction). Thus, nodulated plants maintained higher stomatal conductance under salt stress, in parallel with their improved water status (Figure 3B).

**Figure 3:**
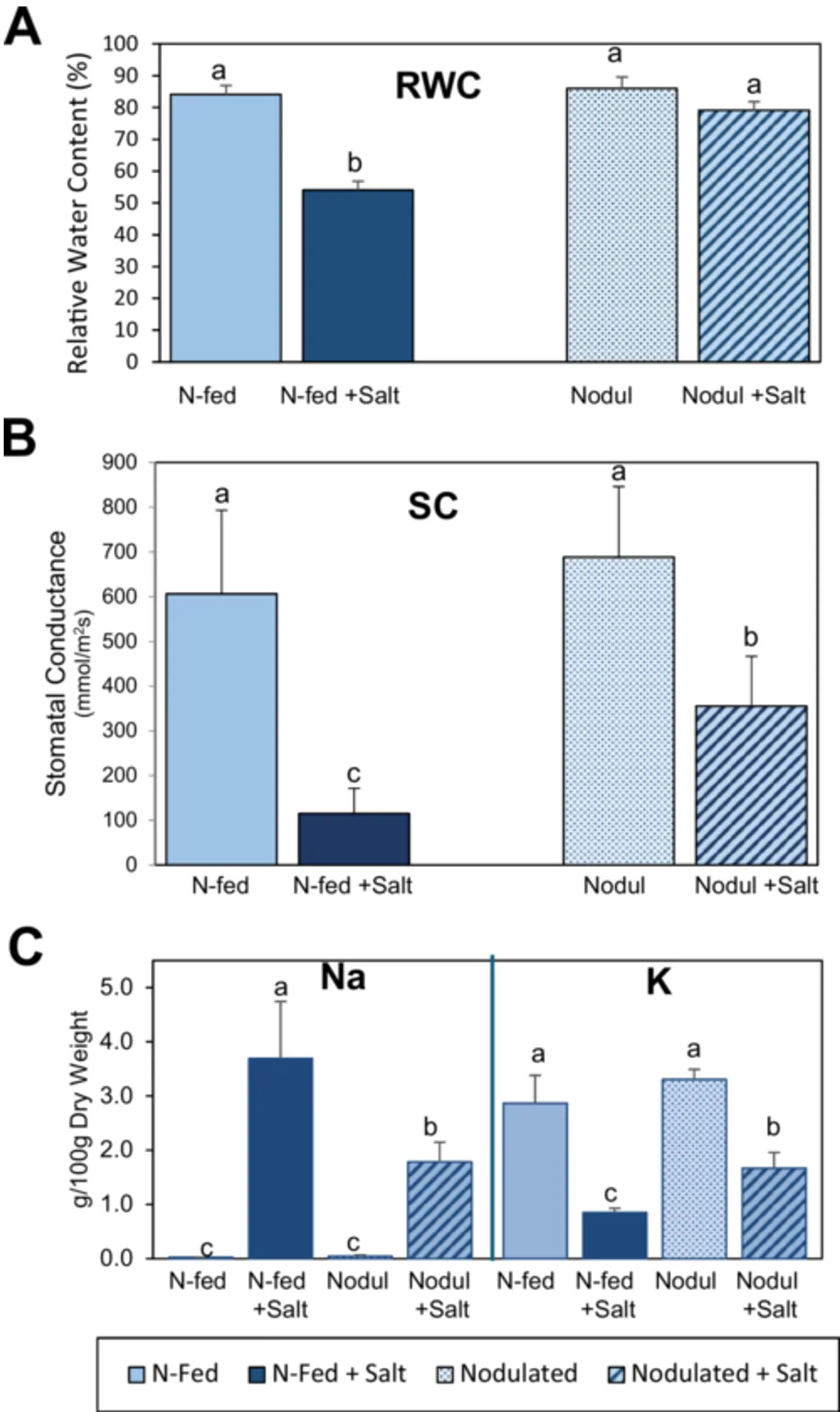
Effect of salt stress and N source on stomatal conductance, relative water content (RWC) and Na‐K levels in vetch plants. (A) RWC of greenhouse‐grown plants under the indicated conditions was measured at 11 days after salt treatment. (B) Stomatal conductance values measured at 11 days after salt treatment. (C) Na and K contents (g 100 g^−1^ DW) in the shoot under the indicated conditions were measured at 11 days after salt treatment. At least three independent experiments were done. *n* = 8 plants each. Values are means ± SD. Letters indicate significant differences (ANOVA and Tukey HSD test *p* < 0.05). [Color figure can be viewed at wileyonlinelibrary.com]

To determine whether differences in water status and stomatal behaviour were associated with changes in ion balance, we quantified Na^+^ and K^+^ levels. Under salinity, as expected, non‐nodulated N‑fed plants accumulated up to 133‑fold more Na, whereas nodulated plants accumulated only 39‑fold more. This Na increase was associated with a marked reduction in K content, which decreased by 70% in N‑fed plants but only 50% in nodulated plants. As a result, the Na^+^/K^+^ ratio was strongly disrupted in N‑fed plants, while nodulated plants maintained a considerably more favourable ionic balance under salt stress (Figure 3C).

Pea plants displayed the same physiological trends, with nodulated individuals maintaining higher RWC, smaller reductions in stomatal conductance, lower Na accumulation, better K retention, and a more favourable Na^+^/K^+^ ratio than N‑fed controls under salinity (Supporting Information S2: Figure S2).

To further investigate the mechanisms underlying the enhanced salt tolerance observed in nodulated plants, we examined several key biochemical markers associated with osmotic adjustment, oxidative stress, and photosynthetic performance, including proline levels, anthocyanin accumulation, lipid peroxidation, and chlorophyll degradation.

Salt stress triggered a significant increase in proline levels in both nodulated and non‐nodulated (N‐fed) plants. However, the level of this response differed markedly between treatments. N‐fed plants exhibited a strong induction of proline accumulation, consistent with a higher degree of osmotic stress. In contrast, nodulated plants displayed only a moderate increase in proline content (Figure 4A).

**Figure 4:**
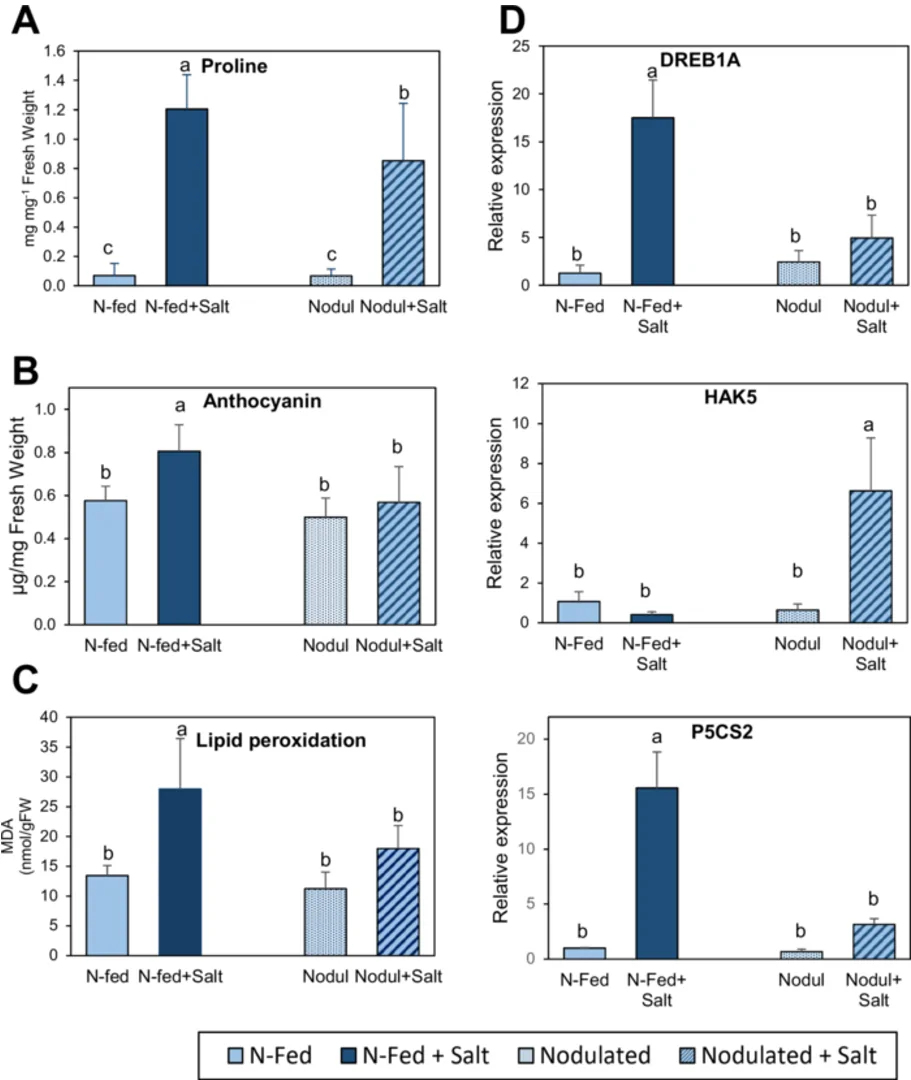
Effect of salt stress and N source on biochemical and molecular stress‐markers in vetch plants. Free proline content (A), anthocyanin level (B), lipid peroxidation assay by malondialdehyde (MDA) relative measurement (C). Expression levels of the indicated stress response genes (D) under the indicated treatments were determined by real‐time RT‐PCR analysis using GADPH as the standard for normalisation. Measurements were done at 11 days after salt treatment. At least three independent experiments were done. *n* = 8 plants each. Values are means ± SD. Letters indicate significant differences (ANOVA and Tukey HSD test *p* < 0.05). [Color figure can be viewed at wileyonlinelibrary.com]

Anthocyanins often accumulate under abiotic stress as part of the antioxidant defence system. In our assays, salinity induced a strong accumulation of anthocyanins in N‐fed plants, consistent with elevated oxidative stress. Importantly, under salt stress conditions, anthocyanin levels were significantly lower in nodulated plants compared to N‐fed plants (Figure 4B).

To evaluate oxidative damage, we measured MDA, a widely used indicator of membrane lipid peroxidation. Salt stress caused a pronounced increase in MDA levels in N‐fed plants, reflecting elevated ROS accumulation and membrane destabilisation. In contrast, nodulated plants showed lower MDA levels under salinity (Figure 4C), with the salt‐induced increase in MDA being significantly lower than in N‐fed plants. Chlorophyll degradation is an indicator of salt‑induced damage to the photosynthetic machinery. To examine whether rhizobial symbiosis helps preserve photosynthetic function, we measured total chlorophyll content in nodulated and N‑fed plants exposed to salinity (Supporting Information S2: Figure S6). Salt stress reduced chlorophyll levels in both groups. Although the differences between nodulated and non‑nodulated plants were not statistically significant, the trend was consistent across experiments: N‑fed plants repeatedly showed a stronger decline in chlorophyll a and b, total chlorophyll and a/b ratio, whereas nodulated plants tended to retain higher pigment levels.

To assess if stress molecular markers were expressed differently in nodulated vs N‐fed plants, we examined the expression of several salt stress or salt tolerance response genes, including the *DREB1A* TF, Δ‐pyrroline‐5‐carboxylate synthetase (*P5CS2*), a key enzyme in proline biosynthesis, and *HAK5*, a high‐affinity potassium transporter involved in salt tolerance. Under saline conditions, a significant upregulation of *DREB1A* and *P5CS2* genes was observed in N‐fed plants. Nonetheless, nodulated plants exhibited a substantially attenuated induction. In contrast, HAK5 expression was induced by salt stress only in nodulated plants (Figure 4D).

A similar biochemical pattern was observed in pea. Under salinity, proline, anthocyanins, MDA (Supporting Information S2: Figure S5D–F) and chlorophylls (Supporting Information S2: Figure S5G–I) displayed response patterns very similar to those observed in vetch. Despite species‐specific differences in absolute values, the general direction of the response was conserved. Importantly, under salt stress conditions, proline and MDA levels showed statistically significant differences between nodulated and N‐fed plants, whereas differences in anthocyanins and chlorophyll‐related parameters did not reach statistical significance and are therefore described as trends. Accordingly, nodulated plants displayed milder changes (lower accumulation of proline and MDA, together with a tendency to retain higher chlorophyll levels and lower anthocyanin accumulation), whereas N‐fed plants exhibited more pronounced alterations. Similarly, in pea, the induction of stress‐marker genes, including dehydrins, LEA proteins, and DREB TFs, appeared to be attenuated in nodulated plants under salt stress (Supporting Information S2: Figure S5J–L).

### 3.4 Transcriptomic Analysis of Differentially Expressed Genes (DEGs) in Response to Salt Stress

The transcriptomic profiles of both shoot and root tissues reveal the coordinated mechanisms through which common vetch responds to salt exposure. Previous comparative transcriptomic analyses have already shown that the molecular pathways activated by salt stress differ markedly between vetch shoots and roots (Lin et al. 2022). To determine whether rhizobial symbiosis modifies the nature of this response, a de novo transcriptome analysis was performed on nodulated and nitrogen‐fed common vetch under control and salt stress conditions in shoot and root. The Supporting Information S2: Figure S2, and Supporting Information S1: Tables S1–S3 and Methods sections provide comprehensive information on assembly, statistics, annotations, and additional results of transcriptomic data. The comparative transcriptomic analysis of shoot (Figure 5) and root (Figure 6) tissues revealed pronounced differences in the molecular response to salt stress depending on the nitrogen source (N‑fed vs. nodulated plants). Both tissues exhibited substantial transcriptional changes upon exposure to salinity, and these responses were not only general but also highly tissue‑specific and nitrogen‑source‑dependent. These genome‐wide analyses were performed in vetch, whereas in pea only targeted expression analyses of selected stress‐responsive genes were conducted.

**Figure 5:**
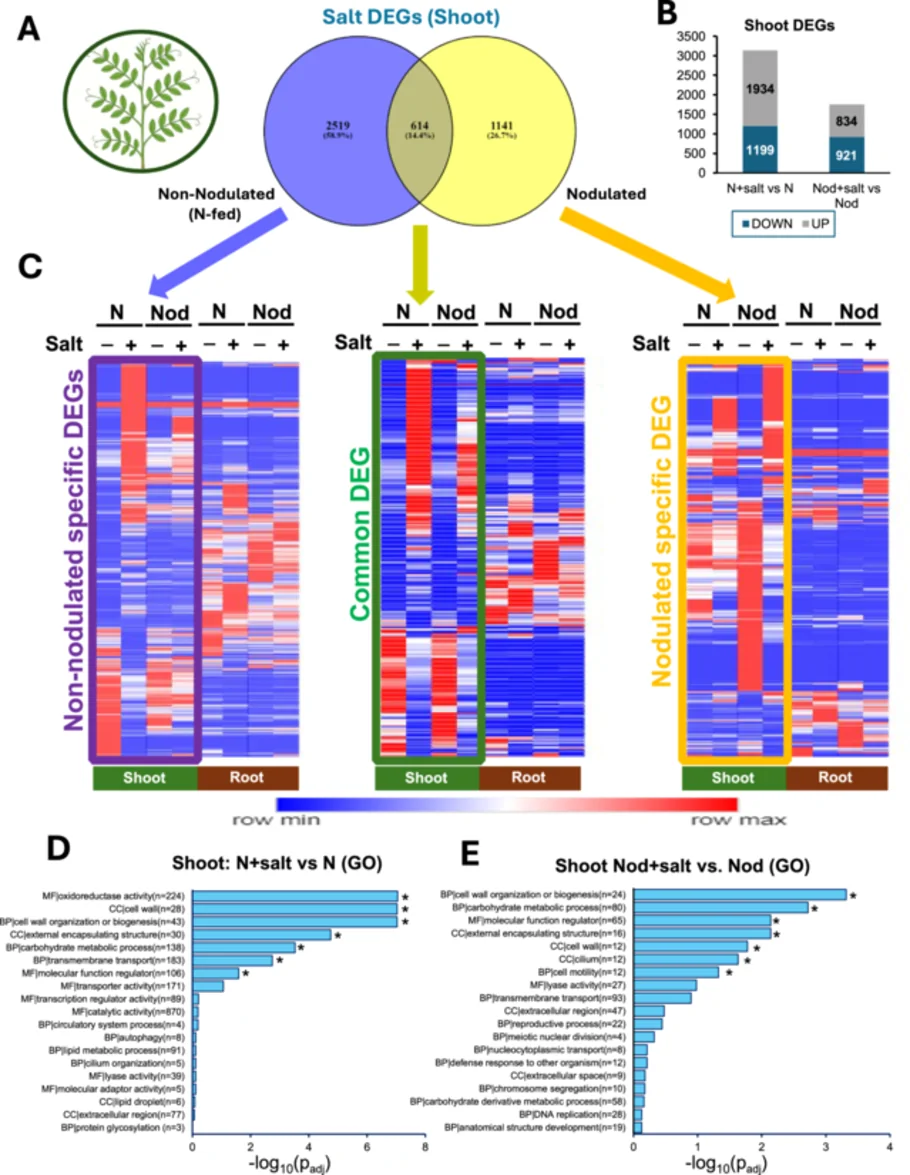
Analysis of differentially expressed genes (DEGs) in response to salt stress in vetch shoot. (A) Venn diagrams showing overlap among DEGs in nodulated versus non‐nodulated (N‐fed) plants under salt stress in vetch shoot. (B) DEG comparison under salt stress of nodulated and N‐fed plants showing up‐ or down‐regulated genes. (C) Hierarchical clustering heatmap visualisation of DEGs based on the expression pattern of salt stress response exclusively in nodulated and non‐nodulated (N‐fed) vetch or commonly deregulated. Colours indicate Z‐score. DEGs enclosed in the coloured rectangles illustrate shoot‐specific deregulation patterns, and the expression profiles of these same genes in roots are shown on the right within the same heatmaps for comparison. (D, E) Enriched gene ontology (GO) terms of DEG genes in vetch plants exposed to salt stress in N‐fed (D) and nodulated conditions (E). BP: biological process, CC: cellular component, MF: molecular function. n indicates gene number. Asterisks indicate significant enrichment of GO terms *p*_adj_ < 0.05. [Color figure can be viewed at wileyonlinelibrary.com]

**Figure 6:**
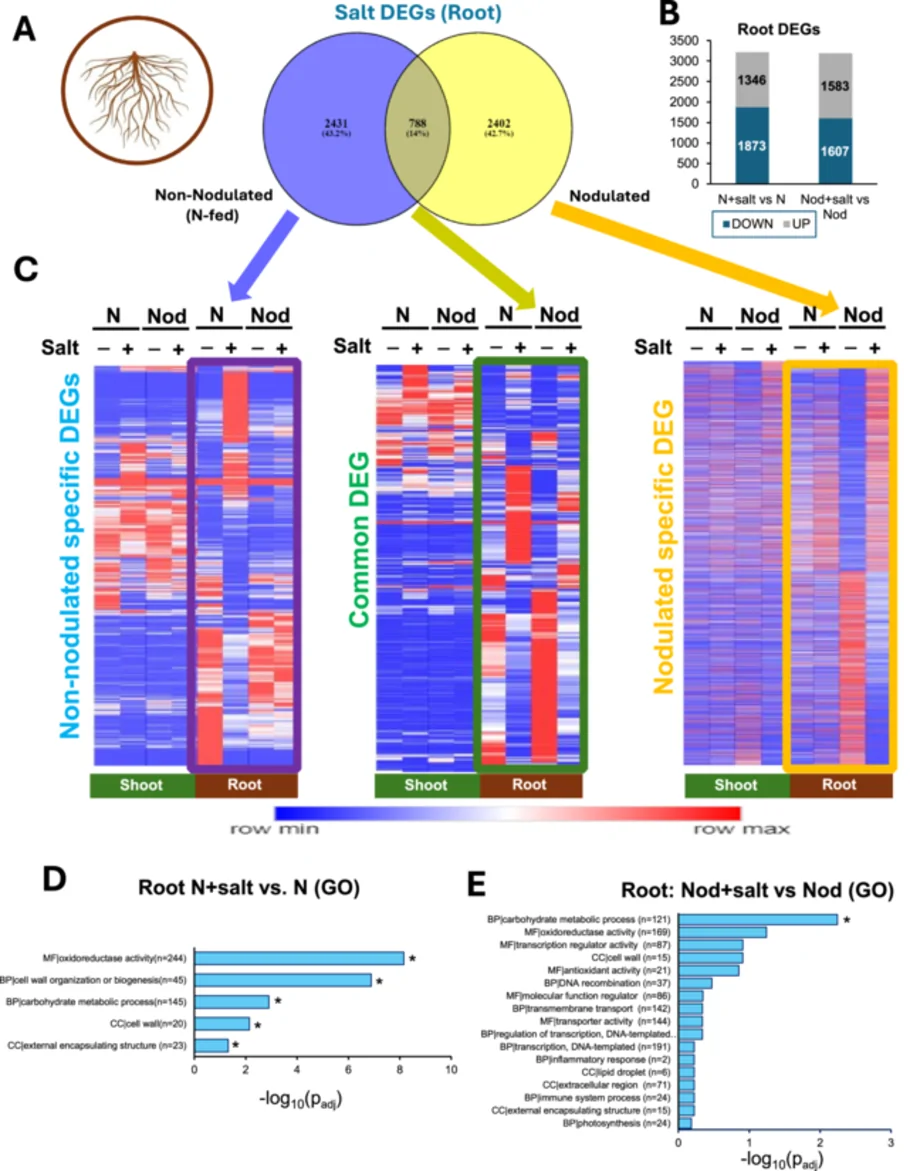
Analysis of differentially expressed genes (DEGs) in response to salt stress in vetch root. (A) Venn diagrams showing overlap among DEGs in nodulated versus non‐nodulated (N‐fed) plants under salt stress in vetch root. (B) DEG comparison under salt stress of nodulated and N‐fed plants showing up‐ or down‐regulated genes. (C) Hierarchical clustering heatmap visualisation of DEGs based on the expression pattern of salt stress response exclusively in nodulated and non‐nodulated (N‐fed) vetch or commonly deregulated. Colours indicate *Z*‐score. DEGs enclosed in the coloured rectangles illustrate root‐specific deregulation patterns, and the expression profiles of these same genes in shoots are shown on the left within the same heatmaps for comparison. (D, E) Enriched gene ontology (GO) terms of DEG genes in vetch plants exposed to salt stress in N‐fed (D) and nodulated conditions (E). BP: biological process, CC: cellular component, MF: molecular function. n indicates gene number. Asterisks indicate significant enrichment of GO terms *p*_adj_ < 0.05. [Color figure can be viewed at wileyonlinelibrary.com]

Thus, in N‑fed plants, a total of 3133 DEGs in shoots (Figure 5A,B) and 3219 in roots were identified under salt stress (Figure 6A,B). In nodulated plants, 1755 DEGs (Figure 5A,B) were detected in the shoot (3190 in roots; Figure 6A,B). To determine whether the salinity‐responsive genes were common or specific to each nitrogen condition, DEGs were classified into three categories: shared, N‐fed‐specific, and nodulation‐specific. As shown in Figures 5A–C and 6A–C, 614 transcripts in shoots and 788 transcripts in roots were commonly deregulated in both N‐fed and nodulated plants under salt stress.

Focusing on shoot tissues, 2519 DEGs were specifically deregulated in nodulated vetch and 1141 DEGs were specifically deregulated in N‑fed vetch (Figure 5A,C), demonstrating marked condition‑specific transcriptional programs. To evaluate their functional relevance, we performed GO enrichment analyses (Figure 5D,E). In shoots, the shared/common DEGs exhibited enrichment for BPs related to cell wall organisation, carbohydrate metabolism, and transmembrane transport, while enriched CC categories included cell wall, extracellular region, and external encapsulating structure. However, nodulation‑specific enrichments were also evident. In contrast, N‐fed plants showed enrichment of stress‐related metabolic and redox processes, consistent with a strong stress response. Nodulated plants instead showed enrichment in processes related to cell division and response to other organisms. Overall, the transcriptomic data revealed both shared and nitrogen‑source‑specific sets of differentially regulated genes, with patterns differing between shoots and roots and between N‑fed and nodulated plants.

A similar pattern was observed in roots, where salinity also triggered marked transcriptional differences between nodulated and N‐fed plants. In this tissue, a total of 788 DEGs were shared between the two nitrogen conditions (Figure 6A–C). However, we also identified two large sets of nitrogen source‐specific DEGs: 2402 deregulated in nodulated roots, and 2431 genes specific to N‑fed roots, highlighting a strong modulation of root transcriptional programs by the nitrogen source. Shared DEGs were enriched in general metabolic and transport processes, representing the core transcriptional response to salinity. Enriched CC categories included cell wall, extracellular region, and external encapsulating structure, while MF terms were dominated by oxidoreductase activities. In contrast, several GO categories were enriched specifically in nodulated roots, pointing to processes uniquely influenced by symbiotic nitrogen fixation. These included BP terms related to transcriptional regulation and transmembrane transport, and MF terms associated with transcriptional and regulatory activity, antioxidant functions, and more diverse regulatory capacities. Complete and detailed GO enrichment results, including specific GO categories, associated pathways, and statistical significance values, are provided in Supporting Information S1: Table S5. Several salt‑responsive DEGs encoded enzymes involved in proline metabolism, such as Δ‑pyrroline‑5‑carboxylate synthetase (P5CS2), as well as numerous cation transporters and antiporters, several dehydrins, and DREB1A. Additionally, numerous members of the WRKY TF family were strongly induced in both shoots and roots under both nitrogen regimes. In contrast, in shoots, certain genes, including DREB1B, the aquaporins TIP1‑like, ethylene‑responsive ERF TFs, various peroxidases, and the ammonium transporter AMT1, were preferentially expressed in nodulated plants. Conversely, other genes showed a stronger response in N‑fed plants, such as key regulators of salt stress, including cation/H^+^ antiporters and DREB1B. The root transcriptomic also included many of these DEGs, with distinct tissue‑specific patterns, showing differences in physiological and molecular responses under salt stress. To further characterise the transcriptional regulatory component of this response, we analysed TF representation among DEGs.

In shoots, 250 TFs were differentially expressed under salinity, including 167 specific to N‐fed plants, 49 to nodulated plants, and 33 shared. In roots, 362 TFs were differentially expressed, of which 173 were specific to N‐fed plants, 134 to nodulated plants, and 55 were shared between conditions. Among these, 88 TFs were shared between shoots and roots, while the remaining TFs displayed tissue‐specific regulation (Figure 7A,B). The most abundant TF families included C2H2, MYB, AP2/ERF, bHLH, WRKY and NAC in both tissues. Enrichment analysis revealed a greater representation of TFs in N‐fed plants compared with nodulated plants. Although some TFs were shared between tissues, distinct family‐specific distributions were observed. In roots, C2H2, bZIP, and bHLH families were preferentially enriched, whereas in shoots MYB, WRKY, AP2/ERF and NAC families showed higher representation. Within these families, several members showed higher representation in N‐fed plants in both tissues (Figure 7C,D). Heatmap analysis of TF expression profiles revealed lower transcriptional variation in nodulated plants compared with N‐fed plants in both shoot and root tissues (Figure 7E,F).

**Figure 7:**
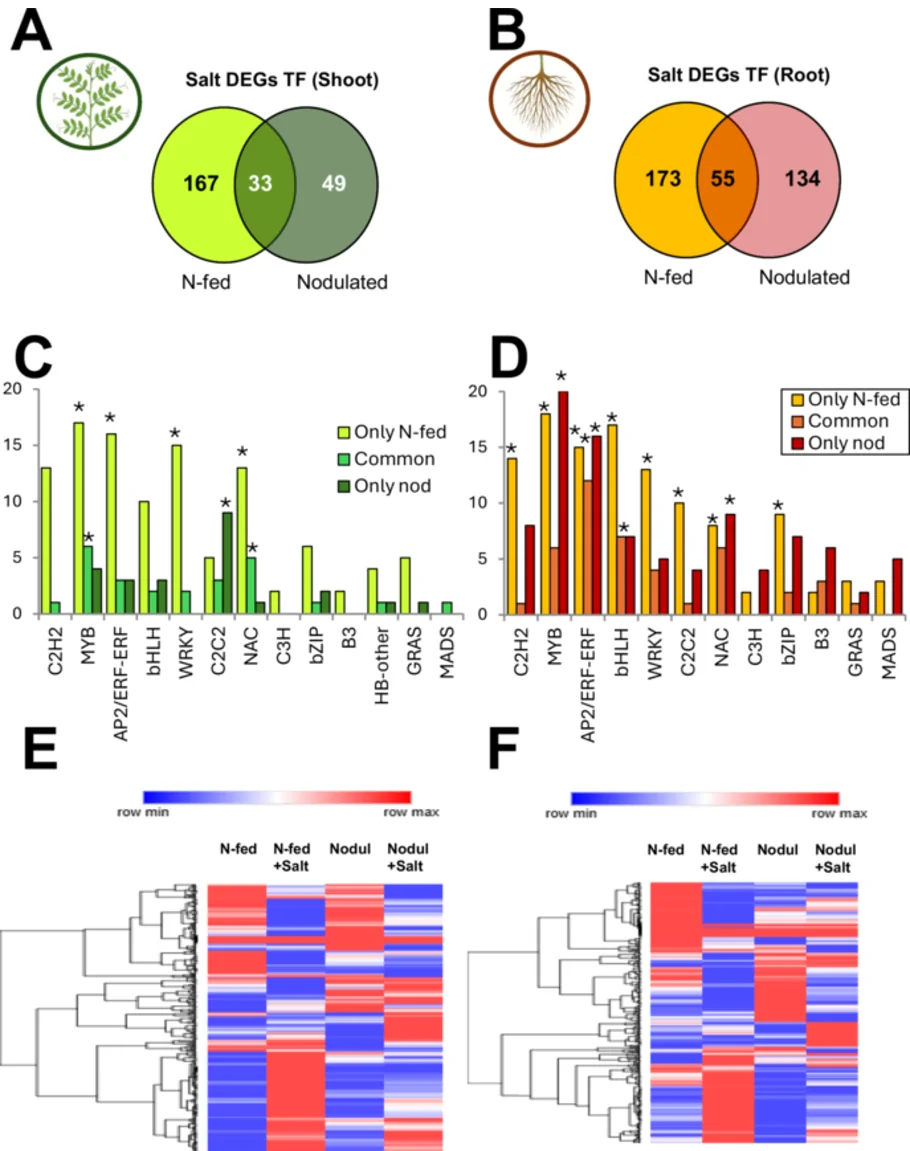
Analysis of differentially expressed transcription factors in response to salt stress. Venn diagram showing the number of differentially expressed TFs of vetch under salinity in the aerial part (A) and root (B). Distribution of differentially expressed TFs by families (C, D) showing N‐fed exclusively enriched TFs, the common TFs and nodulated enriched TFs as indicated. Differences between observed and predicted random coincidences were calculated using a *χ* ^2^ (Chi‐square) binomial test, *p* < 0.05. Asterisks indicate families with significant differences. (E, F) Hierarchical clustering heatmaps of TF genes during salinity response in vetch shoot (E) and root (F). Colours indicate *Z*‐score. [Color figure can be viewed at wileyonlinelibrary.com]

Interestingly, when analysing the effect of nodulation in the absence of salt stress, we observed that a total of 1157 DEGs were identified in shoots when comparing nodulated plants with N‐fed controls, including 742 upregulated and 415 downregulated genes (Supporting Information S2: Figure S9A,B). As expected, the transcriptional effect of nodulation was considerably greater in roots, where 5054 DEGs were detected (3149 upregulated and 1905 downregulated). GO enrichment analyses in shoots (Supporting Information S2: Figure S9C) revealed that, even under non‐stress conditions, symbiosis promotes the activation of BPs related to carbohydrate metabolism, photosynthesis, cell division, and transmembrane transport. Enriched CC categories included intracellular structures such as thylakoids and lysosomes. MF terms were dominated by oxidoreductase activity and hydrolase activity, together with the constitutive expression (under non‐stress conditions) of numerous peroxidases and stress‐related TFs. In roots, GO enrichment analyses showed significant overrepresentation of BP categories associated with carbohydrate metabolism, transmembrane transport, and defence responses, including nodulins and nodule‐specific cysteine‐rich (NCR) peptides, as expected (Supporting Information S2: Figure S9D).

Some of these salinity‐related DEGs were further analysed in their expression levels. Thus, we selected 15 DEGs representing various expression profiles and analysed their expression by quantitative real‐time PCR (RT‐qPCR) using specific primers (Supporting Information S1: Table S4) in nodulated and N‐fed under control or salt conditions, both in the shoot region and the root. These qPCR data were used to ensure the reliability of our RNA‐Seq data. Correlation scatterplots were generated to compare the log_2_‐transformed values of the qPCR data (x‐axis) with those of the RNA‐Seq data (y‐axis). Significant statistical correlation (*r* = 0.84) supports the consistency of the RNA‐seq data (Supporting Information S2: Figure S7).

### 3.5 Metabolic and Ionomic Characterisation of Salt‐Response Traits

To further dissect the physiological advantage conferred by rhizobial symbiosis under salt stress, we performed ionomic analysis (ICP–OES) and untargeted metabolomic profiling (^1^H‐NMR) on nodulated and N‐fed vetch plants under salt treatment.

Ionomic profiling in *V. sativa* demonstrated that salt stress, as expected, led to a marked accumulation of Na, while also causing widespread disruptions in the concentrations of several other mineral nutrients (Figures 3C and 8B; Supporting Information S1: Table S6A). In particular, significant reductions were observed for B, Ca, Fe, K, S and P. Notably, these decreases were generally less pronounced in nodulated plants, with the most attenuated effects detected for B, Ca and P. A PCA performed on the concentrations of the 17 quantified elements revealed that the first two components accounted for a substantial proportion of the total variance: PC1 explained 49.96%, and PC2 explained 19.60%, together capturing 69.6% of the overall variation in the ionomic dataset (Figure 8A). Along PC1, salinity stress drove samples strongly toward the negative axis, a shift associated with a marked increase in Na and concomitant decreases in K, P and Ca. In contrast, nodulated plants (Nod) clustered toward positive PC1 values, showing a general tendency toward higher K, P, Ca and C. Along with PC2, treatments exhibited contrasting ionomic adjustments under salinity. Samples from N‑fed vetches under salinity shifted toward positive PC2 values, characterised by comparatively higher Zn, Cu, B and Na. Conversely, samples from nodulated vetches under salinity clustered toward negative PC2 values, enriched in Sr and Co and relatively depleted in Zn, Cu and B.

**Figure 8:**
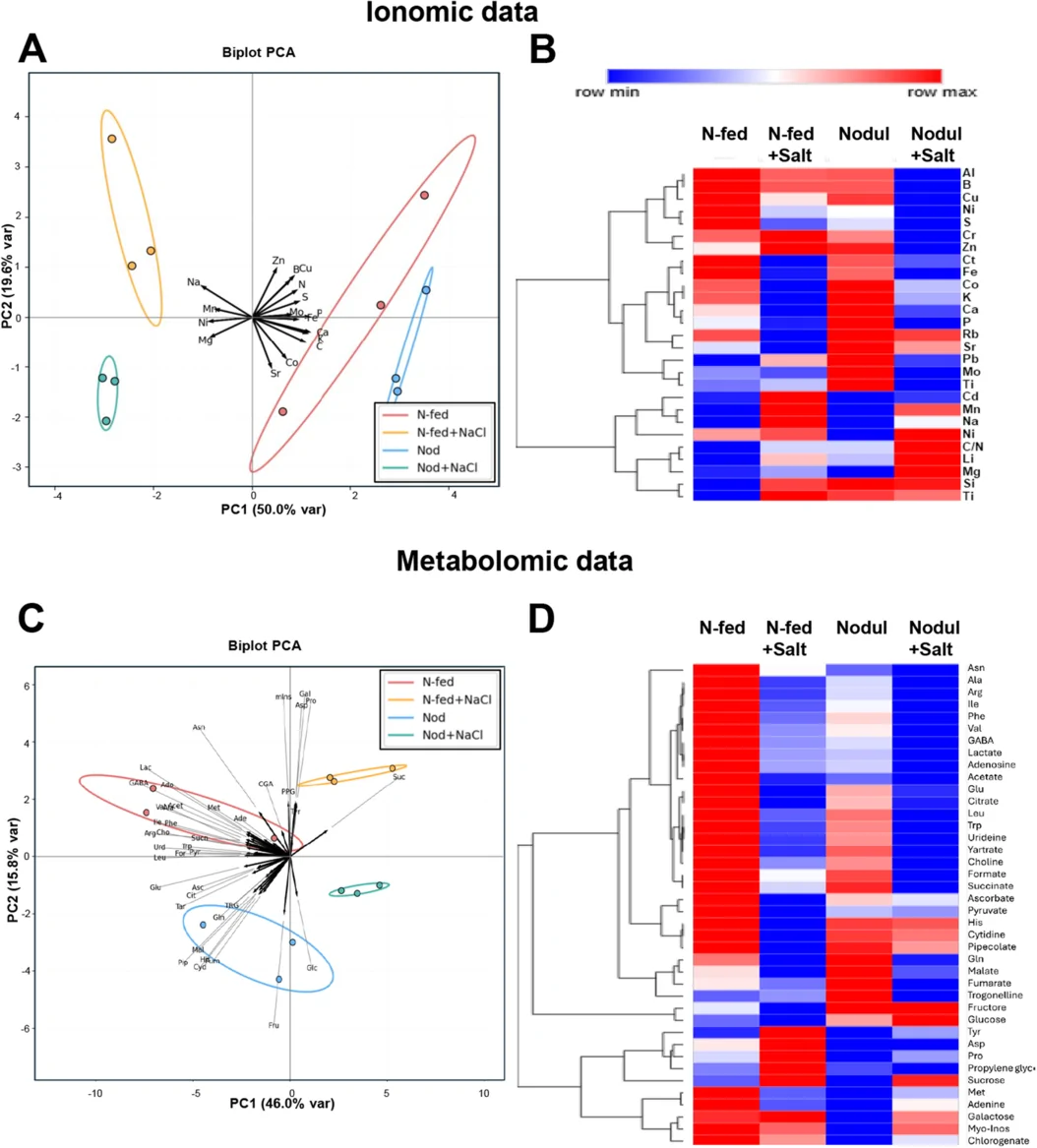
Ionomic and metabolomic profiles of nodulated and N‐fed common vetch plants under salt stress. (A) Multivariate analysis of global ionomic patterns. PC1 and PC2 of the principal component analysis (PCA), including relevant biologically detected elements, are depicted over the x‐axis and the y‐axis, respectively. Dots represent individual sample extracts. (B) Hierarchical clustering heatmaps of ionomic analysis results by ICP–OES of plants under the indicated treatments. (C) Multivariate analysis of global metabolomic patterns. PC1 and PC2 of the principal component analysis (PCA), including relevant biologically detected metabolites, are depicted over the x‐axis and the y‐axis, respectively. Dots represent individual sample extracts. (D) Hierarchical clustering heatmaps of untargeted metabolomic profiling results by ^1^H‐NMR of plants under the indicated treatments. Measurements were done at 11 days after salt treatment. At least three independent experiments were done. *n* = 8 plants each. Values are means ± SD. [Color figure can be viewed at wileyonlinelibrary.com]

A comparable ionomic response was also found in *P. sativum* (Supporting Information S2: Figure S8A,B; Supporting Information S1: Table S6B). The PCA of pea ionomic profiles showed that PC1 and PC2 accounted for 36.6% and 23.7% of the variance, respectively (60.3% cumulative). PC1 separated a nutrient‑rich profile dominated by P, C, B and N, with additional contributions from Fe and S, from a saline‐associated signature driven mainly by Na and Mg. Accordingly, N‐fed and nodulated plants clustered toward positive PC1 values, whereas both saline treatments (N‑fed and nodulated peas) shifted toward negative PC1 values. PC2 contrasted K and Cu (negative) with Zn, Mo and Na (positive), positioning N‑fed+NaCl in the upper region of the biplot, while Nod and Nod + NaCl clustered toward negative PC2 values, showing relatively higher K and Cu.

The metabolomic analysis revealed distinct treatment‑specific profiles supported consistently by both the PCA and the heatmap (Figure 8C,D; Supporting Information S1: Table S7A), observing distinct metabolic signatures associated with symbiosis under salinity, with PC1 explaining 46.0% and PC2 explaining 15.8%. N‑fed plants accumulated high levels of amino acids, whereas N‑fed vetches under stress were dominated by a marked increase in sucrose and other carbohydrates. Nodulated plants showed higher glucose, fructose and glutamine, and exhibited lower accumulation of stress‐related osmolytes (e.g., proline, raffinose) and only moderate metabolic changes, forming a compact cluster with a relatively more balanced carbon‑rich profile under salinity.

In pea, the PCA and heatmap also revealed strong treatment‐specific metabolic profiles (Supporting Information S2: Figure S8C,D; Supporting Information S1: Table S7B). N‐fed peas accumulated high levels of many amino acids and several TCA‐related intermediates. N‐fed peas under salinity also accumulated sucrose and glucose, together with reductions in most amino acids. Nodulated plants showed a distinct metabolic profile enriched in organic acids (fumarate, malate, citrate) and Gln/Glu, while under salt stress symbiosis promotes a pronounced increase in soluble sugars and decrease in amino acids, forming the most distinct cluster under salinity.

## 4 Discussion

The main objective of this study was to understand how rhizobial symbiosis influences the salt stress response in legumes. Our findings show that rhizobial symbiosis strongly improves legume tolerance to salinity, generating consistent physiological and biochemical responses in common vetch and pea. This is consistent with previous studies showing that rhizobial symbiosis can enhance plant performance under different abiotic stresses, mainly drought, in several legumes (Antolín et al. 1995; Álvarez‐Aragón et al. 2023; Amine‐Khodja et al. 2022; Frechilla et al. 2000; Lodeiro et al. 2000; Staudinger et al. 2016), and that rhizobium‐legume interactions can also influence early responses to salt stress (Turetschek et al. 2019; Wekesa et al. 2022). A multitude of interconnected systems, operating at the molecular, cellular, biochemical, morphological, and physiological levels, are responsible for this salinity plant response (Xiao and Zhou 2022).

This physiological improvement was accompanied by a biochemical profile characterised by lower osmotic and oxidative stress, along with a more favourable ionic status. Together, these findings suggest that symbiotic nitrogen acquisition is associated with altered stress perception and stress‐response regulatory pathways, thereby mitigating the damage associated with salinity. In this study, we address, for the first time, the molecular pathways underlying root‐shoot communication during salinity stress and symbiosis in *V. sativa*. To support salt detoxification in both organs, salt stress activates several coordinated regulatory networks and pathways that link and synchronise the root and shoot response systems. Importantly, one of the main strengths of this study is its holistic, multi‑level analytical approach, integrating physiological, biochemical, transcriptomic, ionomic, and metabolomic datasets to generate a comprehensive view of symbiotic mitigation under salinity.

Thus, the physiological responses suggest an enhanced ability to regulate transpiration to cope with the osmotic challenge, consistent with the role of water status and stomatal behaviour in salinity tolerance (Munns and Tester 2008; van Zelm et al. 2020). The K^+^/Na^+^ balance results support the idea that symbiosis buffers water relations and ion homeostasis under salinity (Munns and Tester 2008). Biochemical responses further support this mitigation model. The reduced accumulation of stress‑related metabolites, particularly proline and MDA, supports lower activation of osmotic and oxidative stress pathways under salinity. Anthocyanin accumulation followed a similar pattern, showing statistically significant differences in common vetch, whereas in pea these differences did not consistently reach statistical significance and are therefore described as a trend (van Zelm et al. 2020; Hare et al. 1998; Chalker‐Scott 1999; Hasanuzzaman et al. 2020). These findings are consistent with the higher stomatal conductance and improved ion regulation, indicating an overall reduction in stress, thereby preventing an overall reduction in stress that prevents excessive activation of the redox‐response pathway.

These responses are consistent across different species, such as pea and common vetch, indicating that symbiosis provides a general benefit for salt tolerance in legumes, as highlighted in recent reviews on legume salt stress responses (Bouzroud et al. 2023).

This study provides insights into the transcriptional response of shoots and roots in nodulated versus N‑fed vetch plants under salt stress, revealing molecular mechanisms that help explain the enhanced stress‐mitigation response observed in nodulated plants. Importantly, genome‐wide transcriptomic analyses in this study were conducted exclusively in vetch. Therefore, conclusions regarding transcriptional changes, GO enrichment and TF dynamics are restricted to this species. However, gene expression analysis by RT‐qPCR in pea, using selected stress‐responsive marker genes, suggests a similar response. It is important to note that this study is based on integrative multi‐omics analyses and does not include direct functional validation of the identified pathways or genes. Therefore, the observed changes should be interpreted as associations supported by convergent evidence across datasets rather than as evidence of direct causal relationships, which will require future functional validation. In vetch, salt stress was associated with substantial changes in shoots and roots, but the magnitude, composition, and tissue specificity of the response depended on the nitrogen source, which is consistent with the well‐established organ‐specific transcriptional plasticity described in salt‐stressed plants (Zhao et al. 2021). N‑fed plants showed broader transcriptional activation of genes involved in metabolic remodelling and redox processes, suggesting a more extensive and energy‐demanding stress response, a pattern typical of plants experiencing strong ABA‐driven stress signalling (Wang et al. 2020). In nodulated plants, enrichment of processes associated with cell division and biotic interaction signalling suggests that symbiosis modulates stress signals toward growth‑maintaining and microbe‑responsive pathways rather than initiating generalised defence, which aligns with recent evidence showing that symbiotic contexts can shift TF activity toward growth‐supportive modules under stress (Guo et al. 2024). This aligns with the observed physiological buffering: nodulated plants appear to fine‐tune their response, thereby avoiding a metabolically costly stress state. Differences in stress‑related pathways further support this interpretation. In vetch shoots, genes involved in osmolyte metabolism (e.g., P5CS2), dehydrin/LEA families, cation transporters, and AP2/ERF related TFs were responsive under both nitrogen regimes, revealing a conserved transcriptional core of salinity adaptation, as broadly documented for these gene families under ion‐ and osmotic‐stress responses (Bhat et al. 2024; Admas et al. 2025). Nonetheless, nodulated vetch plants preferentially regulated aquaporins (TIP‐like), certain ERF members, peroxidases, and ammonium transporters, while N‐fed vetches exhibited stronger induction of cation/H^+^ antiporters and selected DREB members, a divergence consistent with classic models of Na^+^ exclusion and ROS‐detoxification trade‐offs (Munns and Tester 2008; van Zelm et al. 2020). The shifted balance of transporter and TF regulation in nodulated plants is consistent with finer control of water movement, ROS detoxification, and nitrogen assimilation under salinity, complementing the ionomic signature of better nutrient retention. By comparison, N‑fed plants activate stronger ion‑expulsion and osmotic‑protection responses, showing that they are perceiving more intense stress, in line with the intensified SOS‐pathway and transcriptional activation described in salt‐sensitive contexts (Ali et al. 2023).

Transcriptional regulation is a central component of plant responses to salinity. Our results indicate that a large number of TFs are activated in the root and shoot of vetch in response to salinity, and this response is distinct between these organs, suggesting different mechanisms of transcriptomic regulation between roots and shoots. Importantly, differences between nodulated and N‐fed plants indicate that symbiosis modulates transcriptional regulatory networks rather than simply altering the magnitude of the stress response. These patterns suggest that nodulated plants maintain a more tightly controlled and balanced transcriptional response, whereas N‑fed plants activate broader stress‐related regulatory programs. A similar pattern of TF deregulation has also been reported in nodulated vetch plants under drought stress, suggesting that symbiosis induces a conserved regulatory signature across different abiotic stresses (Álvarez‐Aragón et al. 2023). This regulatory adjustment is consistent with the reduced stress activation observed at physiological and metabolic levels.

Overall, nodulation appears to induce a priming‑like transcriptional state (Mauch‐Mani et al. 2017). Thus, in vetch, the transcriptional patterns observed in the absence of salt stress indicate that nodulation establishes a constitutively altered regulatory profile (Supporting Information S2: Figure S9), characterised by extensive changes in gene expression in both shoots and roots. These baseline transcriptional changes, together with the enrichment of processes linked to primary metabolism, cellular growth, redox regulation, and defence‑related pathways, suggest that symbiotic plants maintain a preconditioned physiological state that may influence their subsequent responses to environmental challenges. Such a transcriptionally primed condition likely reflects the metabolic demands and signalling requirements of the symbiotic interaction itself, providing a framework to understand how nodulated plants integrate endogenous symbiotic cues with external stress signals.

Ionomic data further support this model. In both common vetch and pea, salinity caused a clear ionomic imbalance with strong Na (and Mg) accumulation and decreases in K, P, Ca and B, a pattern broadly consistent with ionomic disruption typically observed under salt stress (Munns and Tester 2008). Nodulated plants maintained improved nutrient balance under salinity, in agreement with the essential roles of K^+^, Ca^2+^ and P in maintaining membrane stability, enzyme function, and osmotic adjustment under salinity (Shabala and Cuin 2008; White 2003). This is aligned with physiological maintenance of water status and reduced oxidative damage and is associated with transcriptomic indications in vetch of efficient transporter regulation under symbiosis. In contrast, N‐fed plants under salinity displayed more depleted nutrient profiles and, in pea, higher Zn and Mo levels, a pattern commonly linked to stress‐driven adjustments in micronutrient transport and homeostasis (Gupta et al. 2016; Grattan and Grieve 1998). Despite salinity reducing both nodule biomass and nitrogenase activity, the nodulated plants still maintained a considerable level of total N and showed only minor shifts in total C and the C/N ratio. These results clarify the relationship between the reduction in nitrogenase activity and the description of a “stable carbon‐nitrogen metabolism” under moderate salt stress. Here, “stable” refers to the maintenance of a relatively balanced carbon and nitrogen status rather than to the absence of changes. In this context, N content remains largely preserved, while carbon levels and the C/N ratio are only moderately affected. Under these conditions, plants do not experience severe N deficiency or C starvation. In contrast, under more severe salt stress conditions, a drastic inhibition of nodulation and nitrogenase activity is observed, leading to a pronounced C/N imbalance (Supporting Information S2: Figure S10).

Importantly, the observed effects are not explained solely by nitrogen availability. Under high nitrogen supply, where nodulation is suppressed, inoculated plants showed responses comparable to non‐nodulated N‐fed plants under both control and salt stress conditions. In contrast, under low nitrogen conditions, non‐inoculated plants displayed clear symptoms of nitrogen deficiency. These observations support that the enhanced salt tolerance observed in nodulated plants is specifically associated with active symbiosis rather than with general nitrogen status (Supporting Information S2: Figure S11).

This indicates that moderate salinity does not significantly disturb the overall nutrient balance in symbiotic plants, consistent with reports that rhizobial symbiosis can stabilise nutrient acquisition and nitrogen economy under saline conditions (Garg and Singla 2004). Overall, the results support that in both vetch and pea, nodulation mitigates the nutritional impact of salinity, preserving key nutrient pools and limiting the micronutrient alterations observed in non‑nodulated, salt‑stressed plants.

Metabolomic data further reinforce the model in which symbiosis mitigates stress. N‐fed plants exposed to salinity exhibited extensive carbohydrate reprogramming, with high sucrose accumulation and broader amino acid reductions, well‐established markers of acute stress that are associated with shifts in metabolism toward osmotic balance and energy supply. This has been widely observed in salt‐stressed plants undergoing metabolic changes (Krasensky and Jonak 2012). In contrast, nodulated plants maintained a compact, carbon‐enriched metabolic profile with higher levels of glucose, fructose, and glutamine and only moderate changes in osmolytes such as proline and raffinose, consistent with reports that symbiosis can stabilise core carbon and nitrogen metabolism under stress (Larrainzar et al. 2009). This pattern suggests that symbiosis helps maintain metabolic homeostasis under salinity, reducing the need for strong osmotic adjustments and allowing the plant to maintain a flexible metabolism. In both vetch and pea, nodulated plants showed consistent retention of organic acids such as malate, fumarate, and citrate, along with elevated Gln/Glu levels, features indicative of sustained TCA‐cycle activity, carbon flux, and nitrogen assimilation, thereby supporting energy balance and reducing‐power reductant supply during stress (Obata and Fernie 2012). The stability of total N, C content and C/N ratio observed in symbiotic plants under salinity aligns with these metabolic patterns, reinforcing the idea that nodulation sustains nutrient availability and metabolic homeostasis even when nitrogenase activity is partially compromised, as supported by broader evidence that biological nitrogen fixation can buffer metabolic perturbations under stress (Udvardi and Poole 2013). Conversely, N‐fed plants under salinity displayed more pronounced metabolic alterations characterised by sugar accumulation, amino acid depletion, and a collapse of central carbon metabolism, patterns frequently described in salt‐sensitive metabolic responses (Li et al. 2017). Overall, the shared responses of vetch and pea show that BNF mitigates the metabolic cost of salinity, stabilising nutrient homeostasis and maintaining a more balanced interplay between carbon and nitrogen metabolism. Despite species‐specific details in metabolite profiles, both legumes converge on the same physiological principle: nodulation enhances tolerance by sustaining nutrient availability, moderating stress‐induced metabolic shifts, and supporting a more functional metabolic state to cope with saline stress.

Taken together, these results support a model in which symbiosis attenuates stress perception and promotes a more balanced physiological and metabolic response under salinity (Maurel et al. 2015; Munns and Tester 2008). Legumes cope with salt stress through a wide range of physiological, molecular, and symbiotic strategies that aim to preserve their productivity under adverse conditions (Bouzroud et al. 2023). From an applied perspective, our findings highlight the potential of rhizobial inoculation as a promising strategy for mitigating salinity stress in legumes, in line with the broad evidence supporting microbial inoculants as effective tools for improving crop performance under abiotic stress (Nadeem et al. 2014). The conserved responses observed in both vetch and pea suggest that the benefits of symbiosis may extend to other legume species. Inoculating legume crops with selected rhizobial strains not only reduces the need for nitrogen fertilisers, promoting more sustainable agricultural practices, but can also lead to substantial yield improvements, providing clearer economic and ecological benefits. In addition to selecting plant genotypes with enhanced salinity tolerance, a key implementation priority is the identification of rhizobial strains that not only nodulate efficiently but also maintain their symbiotic benefits in a stress‐responsive manner. Recent work in common vetch has further shown that plant genotype can strongly influence salt tolerance (Sun et al. 2022) and nitrogen fixation efficiency or nodulation ability (López‐Román et al. 2025), highlighting the importance of matching host genotypes with optimal rhizobial partners. At the breeding and biotechnology level, prioritising transporter and TF nodes identified as differentially regulated under symbiosis, together with markers of ionomic and metabolomic stability, could be a useful strategy for the design of cultivars optimised for saline soils.

This study also has important limitations and directions for future research. Although the influence of rhizobial symbiosis on salinity responses has been examined from multiple physiological, molecular, and metabolic perspectives, we must consider the inherent complexity of this trait. Salinity response and tolerance can vary widely not only among legume species, but also among genotypes within a species, as well as across rhizobial strains with different symbiotic efficiencies, underscoring the need to consider host‐microbe specificity and compatibility when interpreting stress responses. In addition, the nature of salinity itself is heterogeneous: salt stress may result either from irrigation‐induced salt accumulation in soils or from irrigation‐driven salt accumulation, producing distinct temporal osmotic and ionic pressures. Moreover, salinity frequently coincides with drought or heat, combining stress effects and complicating response analyses.

Rather than being controlled by a single master regulator, the enhanced salt tolerance observed in nodulated plants likely results from the coordinated action of multiple physiological and molecular processes. These include changes in water status, ion homeostasis, metabolism, and gene expression across different tissues. Such a multilevel response is consistent with the idea that plant stress tolerance is governed by interconnected networks rather than by a single pathway. In this context, the strength of this study lies in the integration of independent datasets, which together reveal consistent patterns at the whole‐plant level. Our results show that rhizobial symbiosis shifts the salinity response of legumes toward a more efficient and physiologically balanced acclimation strategy. Symbiotic plants sustain a more favourable nutritional status, maintain osmotic and redox balance, and preserve central carbon‐nitrogen metabolism, thereby avoiding the severe metabolic and cellular disruptions typically triggered by salinity. These adjustments support improved homeostasis under stress and ultimately translate into enhanced growth, survival, and productivity in saline environments. Overall, rhizobial symbiosis provides a systems‐level mechanism that strengthens legume tolerance to salinity and represents a promising target for inoculant development, as a salt stress management approach for agronomic optimisation and breeding strategies aimed at stabilising crop performance in increasingly salt‐affected agroecosystems under the current climate change conditions.

## Conflicts of Interest

The authors declare no conflicts of interest.

## Supporting information


Supporting File 1

Supporting File 2

## Data Availability

The data that support the findings of this study are openly available in NCBI as Sequence Read Archive (SRA) at http://www.ncbi.nlm.nih.gov/bioproject/, reference numbers PRJNA1482267 and PRJNA1482462. The raw sequencing data generated in this study have been deposited in the NCBI Sequence Read Archive (SRA) under BioProject accessions PRJNA1482267 and PRJNA1482462. Additional data supporting the findings of this study are available upon request.
