# Supplementary material for: Rhizobial Symbiosis Enhances Salinity Tolerance in Legumes Through Coordinated Physiological and Molecular Responses: Evidence From Common Vetch and Pea
Source: Plant Cell Environ. 2026 Aug 4;49(11):8912–31. doi: 10.1111/pce.70790 (PMC13646694; doi:10.1111/pce.70790)
Supplement: Supplementary file 1 [file PCE-49-8912-s001.pdf]

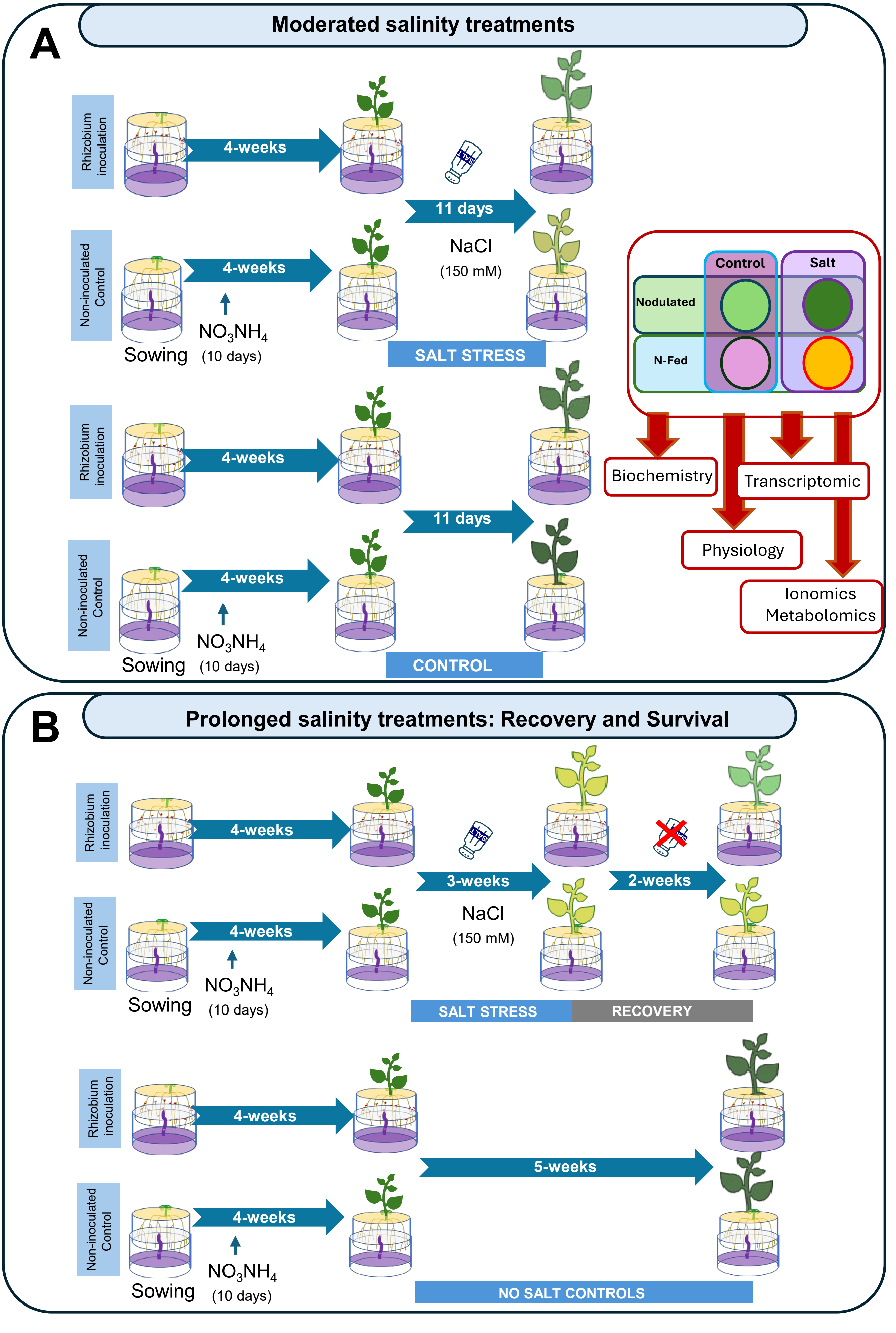

Supplemental Figure S1. Diagram and timeline of the procedures performed for inoculation and moderate **(A)** or prolonged **(B)** salinity treatments. Detail information is included in the materials and methods section.

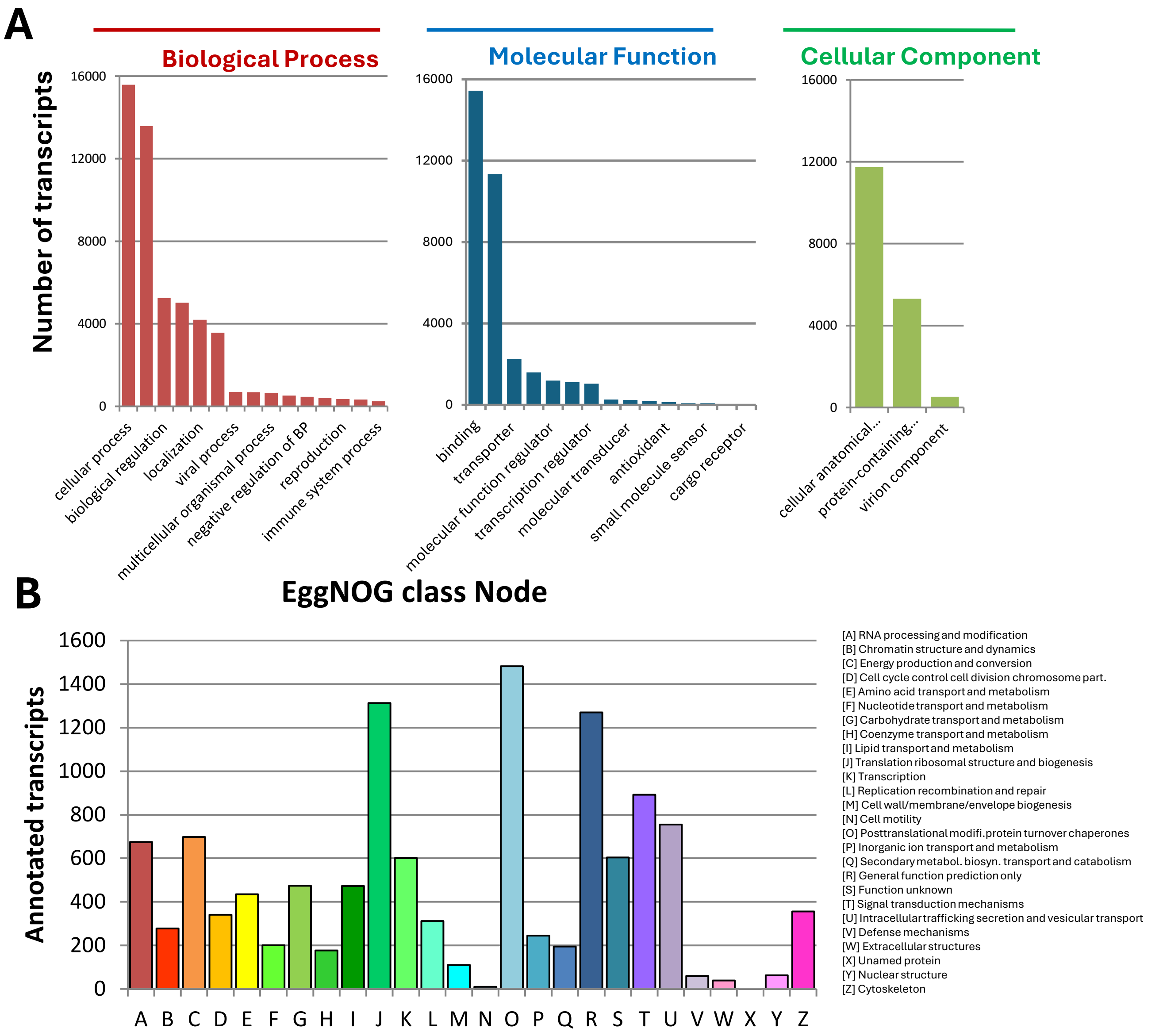

Supplemental Figure S2. Main GO and EggNOG categories of the *de novo* assembled annotated *V. sativa* transcripts. **A.** GO function classification of the annotated transcripts (TOP 15) major categories involved in Biological Process Molecular Function and Cellular Component. **B.** Clusters of orthologous groups (COG) classification of the assembled unigenes into main EggNOG functional categories according to their predicted gene products.

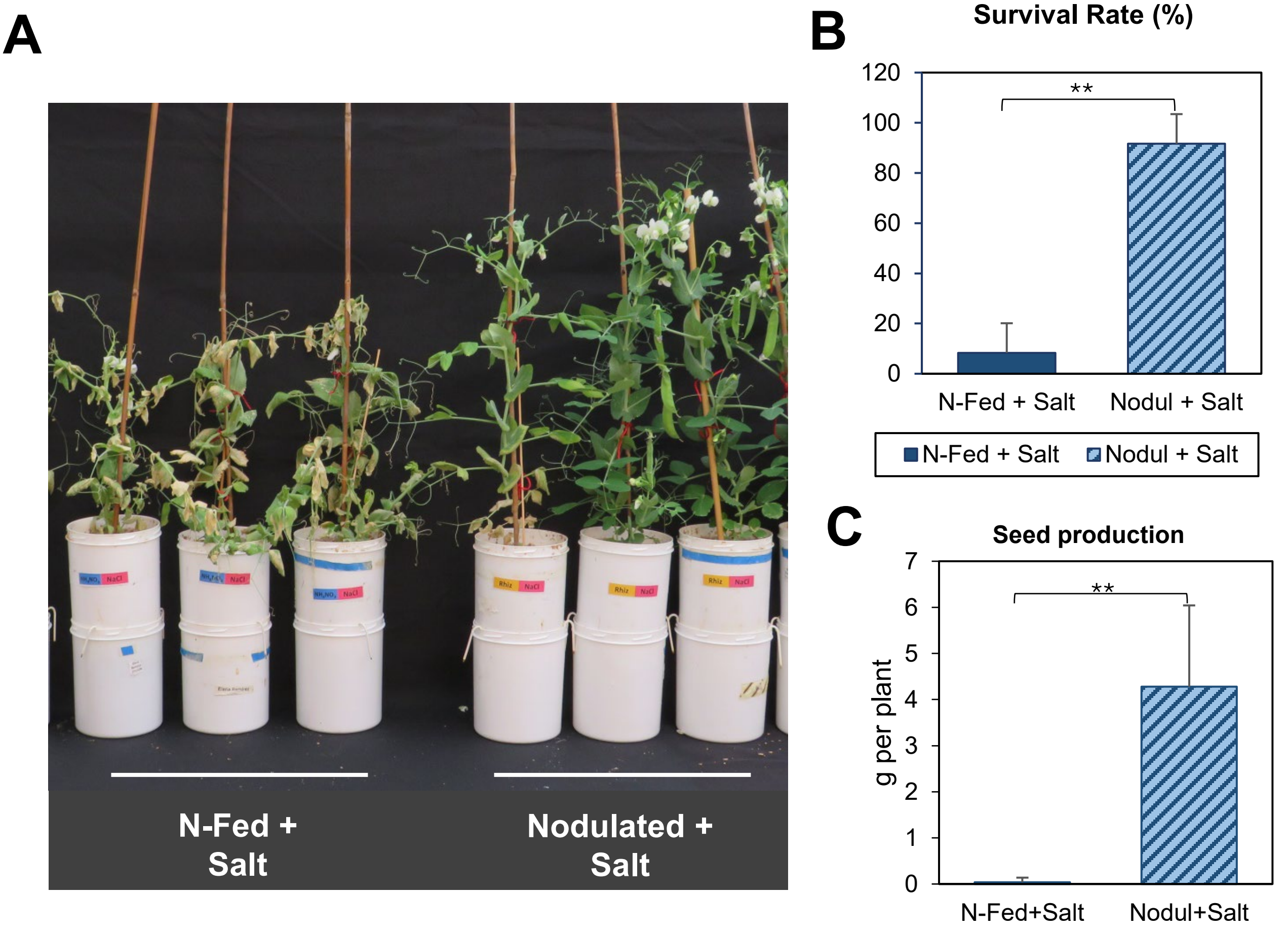

Supplemental Figure S3. Enhanced salt tolerance in nodulated pea plants relative to nitrogen-fertilized plants. A. Four-week-old nodulated and N-fed plants were exposed to salt stress for 3 weeks and then the stress was removed for 8 days. Survival rate (**B**) and seed production (**C**) yield in nodulated and N-fed plants after3 weeks days of salt stress and 23 days of recovery. Student T-test (**) $p < 0.005$. n=12 plants per treatment.

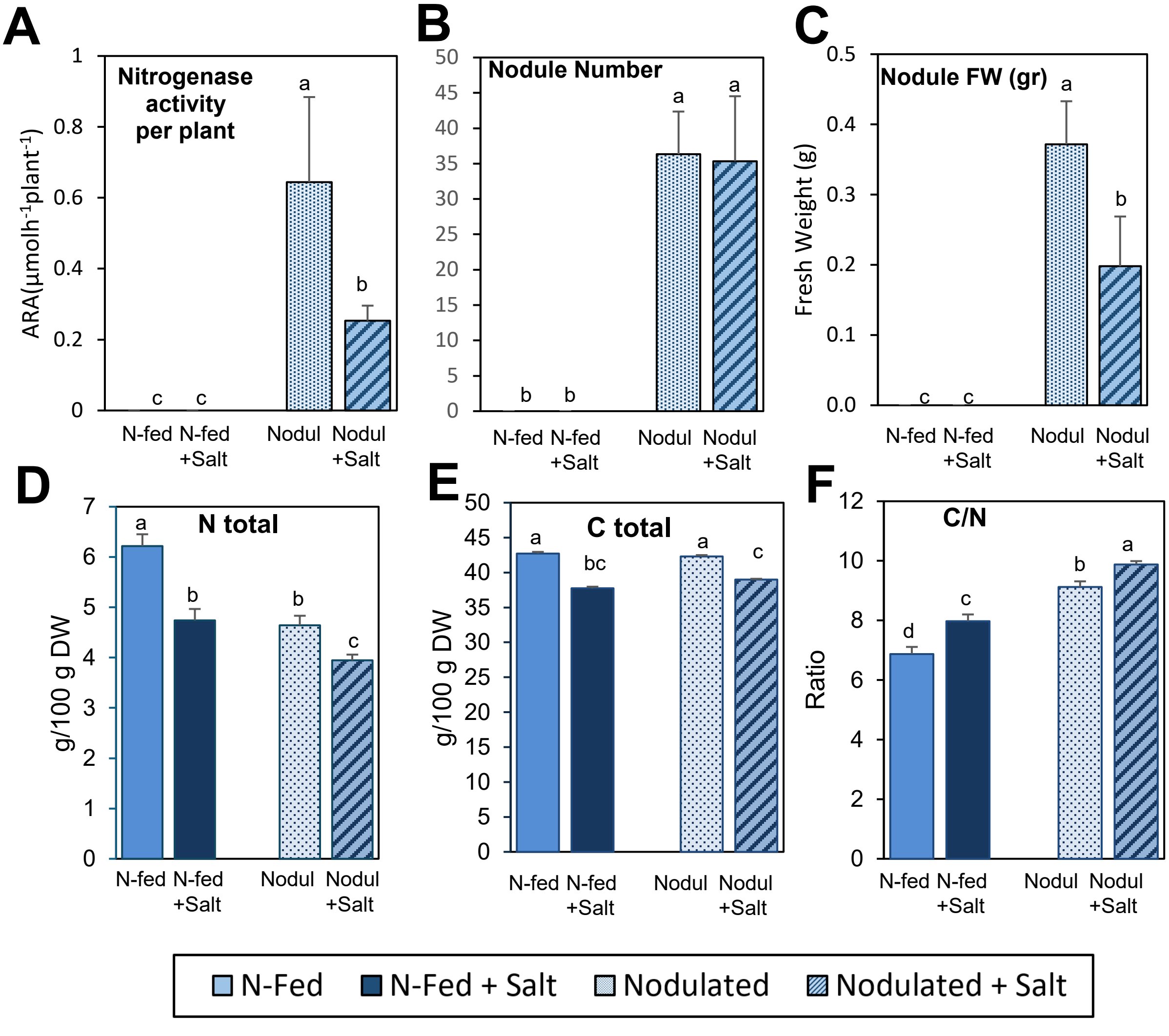

Supplemental Figure S4. Effect of salt on Rhizobium-pea symbiosis. A. Nitrogenase activity per plant by Acetylene Reduction Assay (ARA) method**. B.** Nodule number per plant. **C.** Nodule total fresh weight per plant. Quantification of N (**D**) and C (**E**) content and C/N ratios (**F**) on shoot at indicated conditions. At least three independent experiments were performed (n=6 plants each). Values are means ± sd. Letters indicate significant differences. *p*<0.01 (ANOVA and Tukey HSD test).

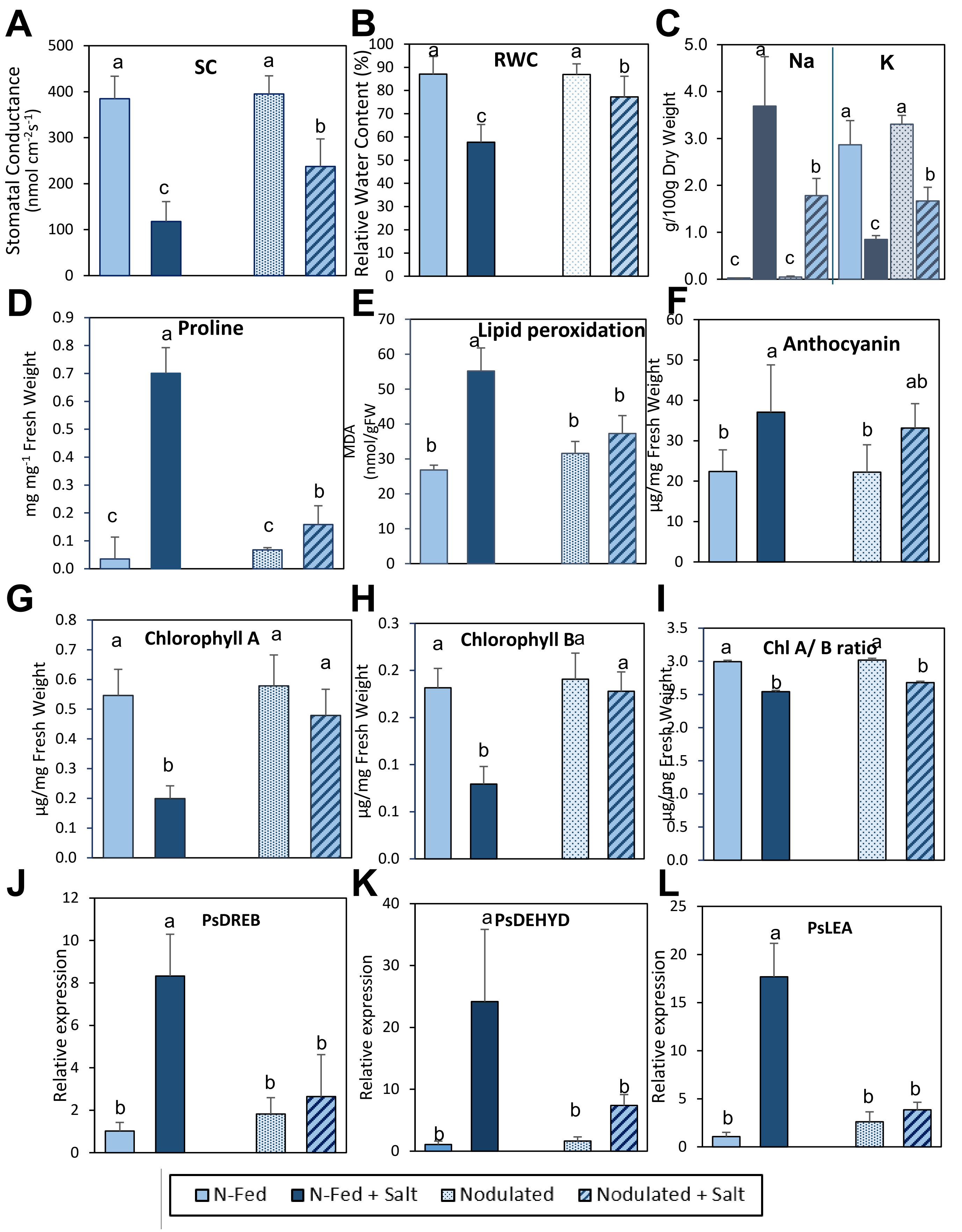

Supplemental Figure S5. Effect of salt stress and N source on physiological, biochemical and transcriptomic parameters in pea plants. **A.** Relative Water Content (RWC) of greenhouse-grown pea plants under the indicated conditions. **B.** Stomatal conductance values measured at 11 days after salt treatment. **C.** Na and K contents (g 100g-1 DW) in shoot under the indicated conditions. Free proline content (**D**), anthocyanin level (**E**), lipid peroxidation assay by Malondialdehyde (MDA) relative measurement (**F**). Chlorophyll a level **(G),** chlorophyll b level **(H),** and chlorophyll a/b ratio **(I).** Expression levels of the indicated stress response genes (**J-L**) under the indicated treatments were determined by real-time RT-PCR analysis using GADPH as the standard for normalization. Measurements were done at 11 days after salt treatment. At least three independent experiments were done. n=8 pea plants each. Values are means ± sd. Letters indicate significant differences (ANOVA and Tukey HSD test $p<0.05$).

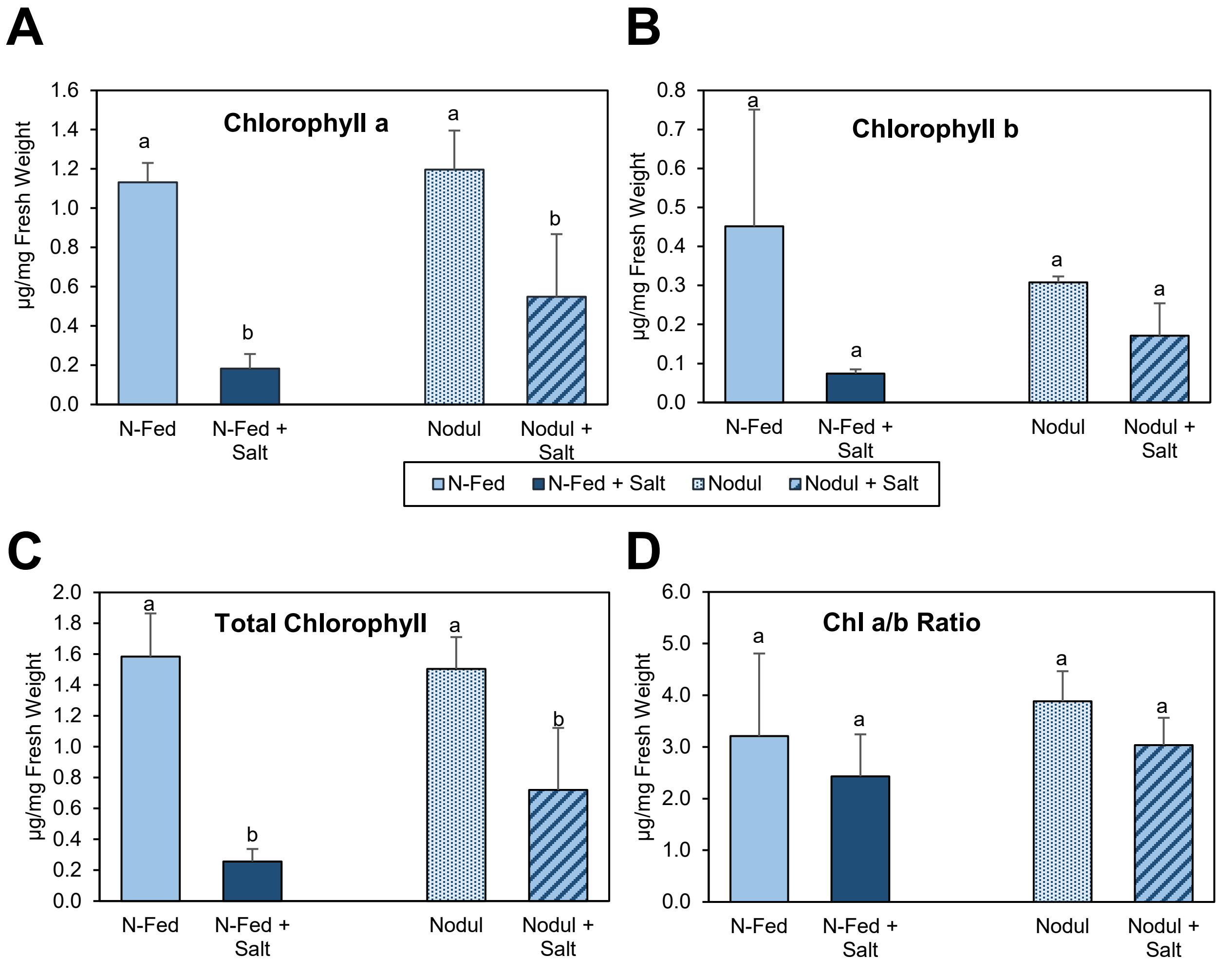

Supplemental Figure S6. Effect of salt stress and N source on photosynthetic pigment content in vetch plants. Chlorophyll a level (A), chlorophyll b level (B), total chlorophyll content (C) and chlorophyll a/b ratio (D). Measurements were done at 11 days after salt treatment. At least three independent experiments were done. n=8 vetch plants each. Values are means ± sd. Letters indicate significant differences (ANOVA and Tukey HSD test $p<0.05$).

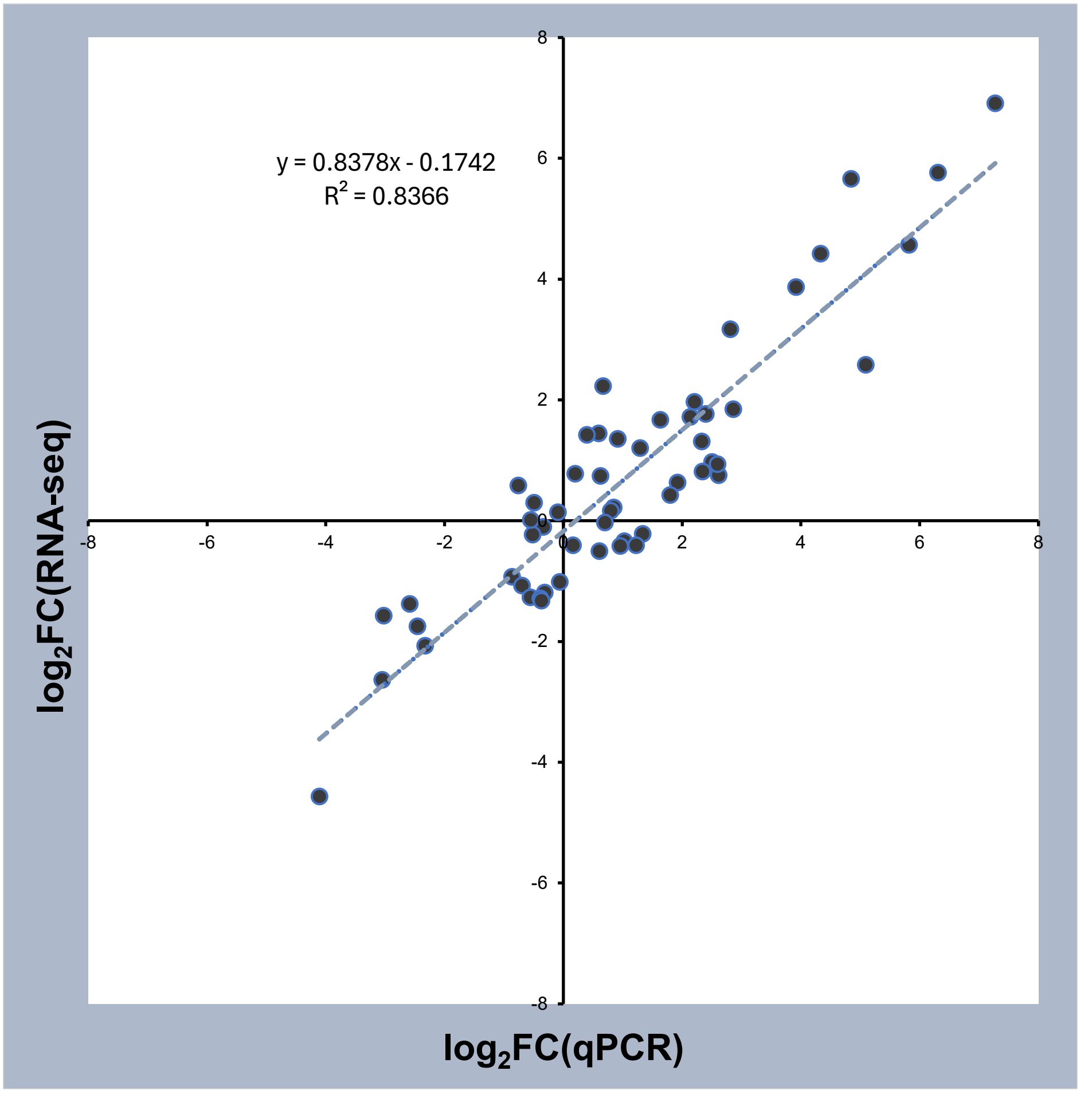

Supplemental Figure S7. Correlation scatterplot for Fold Changes (logarithmic ratios) from RNA-seq experiments and qPCR data of the selected 12 genes tested in inoculated and non-inoculated plants under salt stress and control conditions. Both log 2-fold changes were well correlated (R^2 = 0.84).

Ionomic data

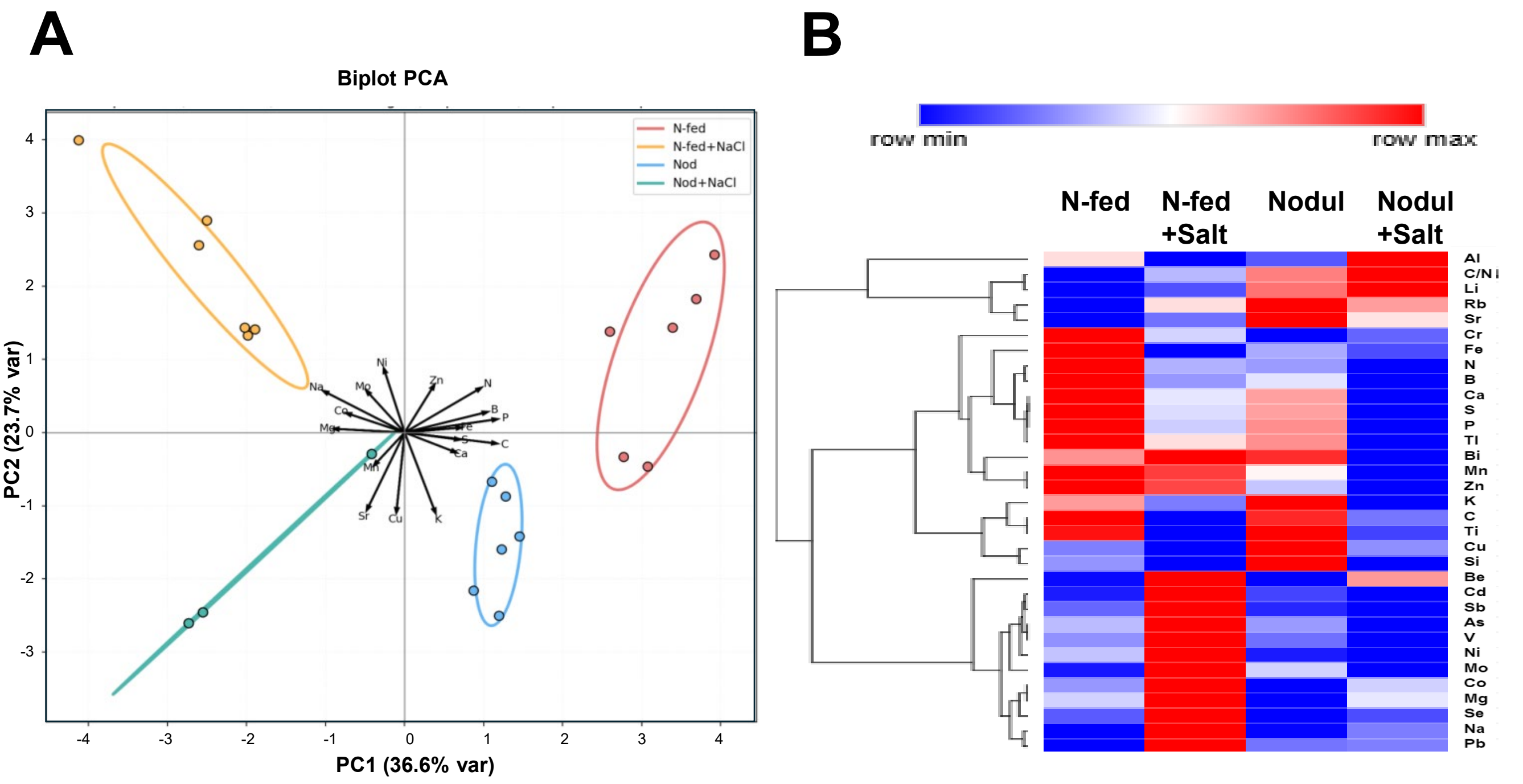

Metabolomic data

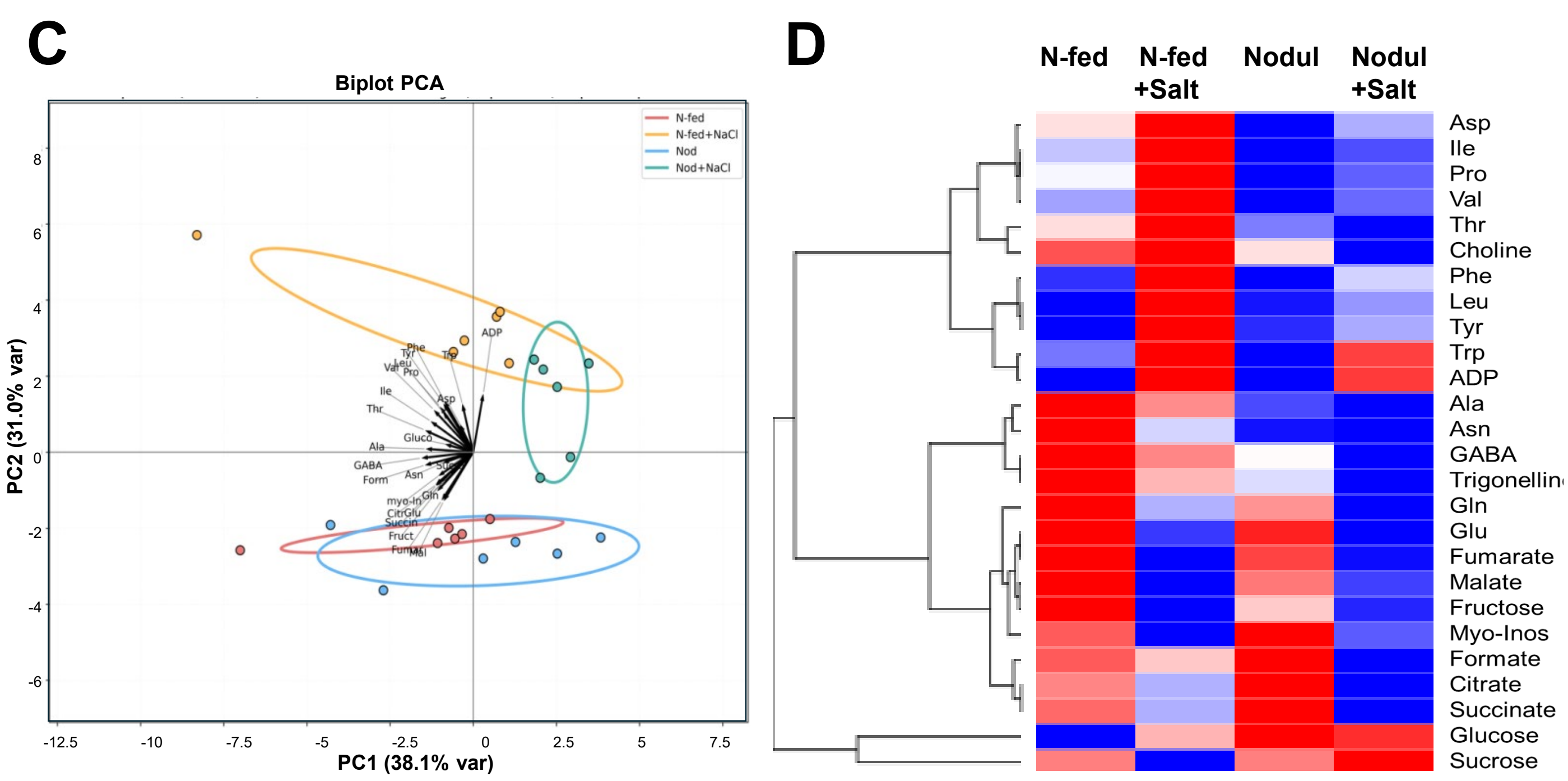

Supplemental Figure S8. Ionomic and metabolomic profiles of nodulated and N-fed common pea plants under salt stress. A. Multivariate analysis of global ionomic patterns. PC1 and PC2 of the principal component analysis (PCA) including relevant biological detected elements are depicted over the *x*-axis and the *y*-axis, respectively. Dots represent individual sample extracts. **B.** Hierarchical clustering heatmaps of ionomic analysis results by ICP–OES of plants under the indicated treatments. **C.** Multivariate analysis of global metabolomic patterns. PC1 and PC2 of the principal component analysis (PCA) including relevant biological detected metabolites, are depicted over the *x*-axis and the *y*-axis, respectively. Dots represent individual sample extracts. **D.** Hierarchical clustering heatmaps of untargeted metabolomic profiling results by 1H-NMR of plants under the indicated treatments. Measurements were done at 11 days after salt treatment. At least three independent experiments were done. n=8 plants each. Values are means ± sd.

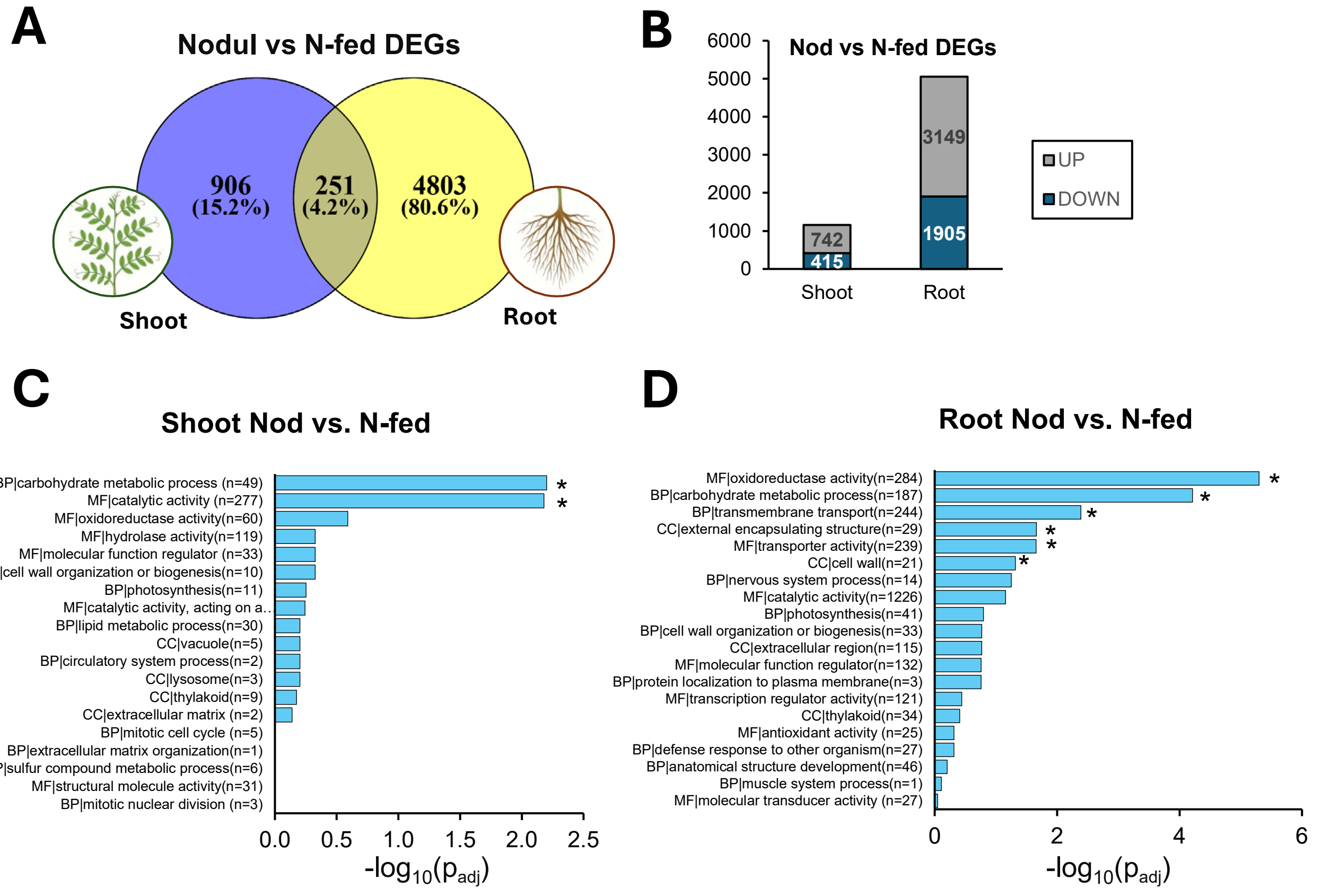

Supplemental Figure S9. Analysis of Differentially Expressed Genes (DEGs) between nodulated and non-inoculated (N-fed) plants. A. Venn diagrams showing overlap among DEGs in nodulated versus non-nodulated (N-fed) plants in vetch shoot and root. **B.** DEG comparison of nodulated and N-fed plants showing up- or down-regulated genes. **C-D**. Enriched gene ontology (GO) terms of DEG genes in nodulated vetch plants versus N-fed conditions in shoot (C) and root (D). BP: Biological Process, MF: Molecular Function, CC:Cellular Component. n indicates gene number. Asterisks indicate significant enrichment of GO terms $p_{adj}<0.05$.

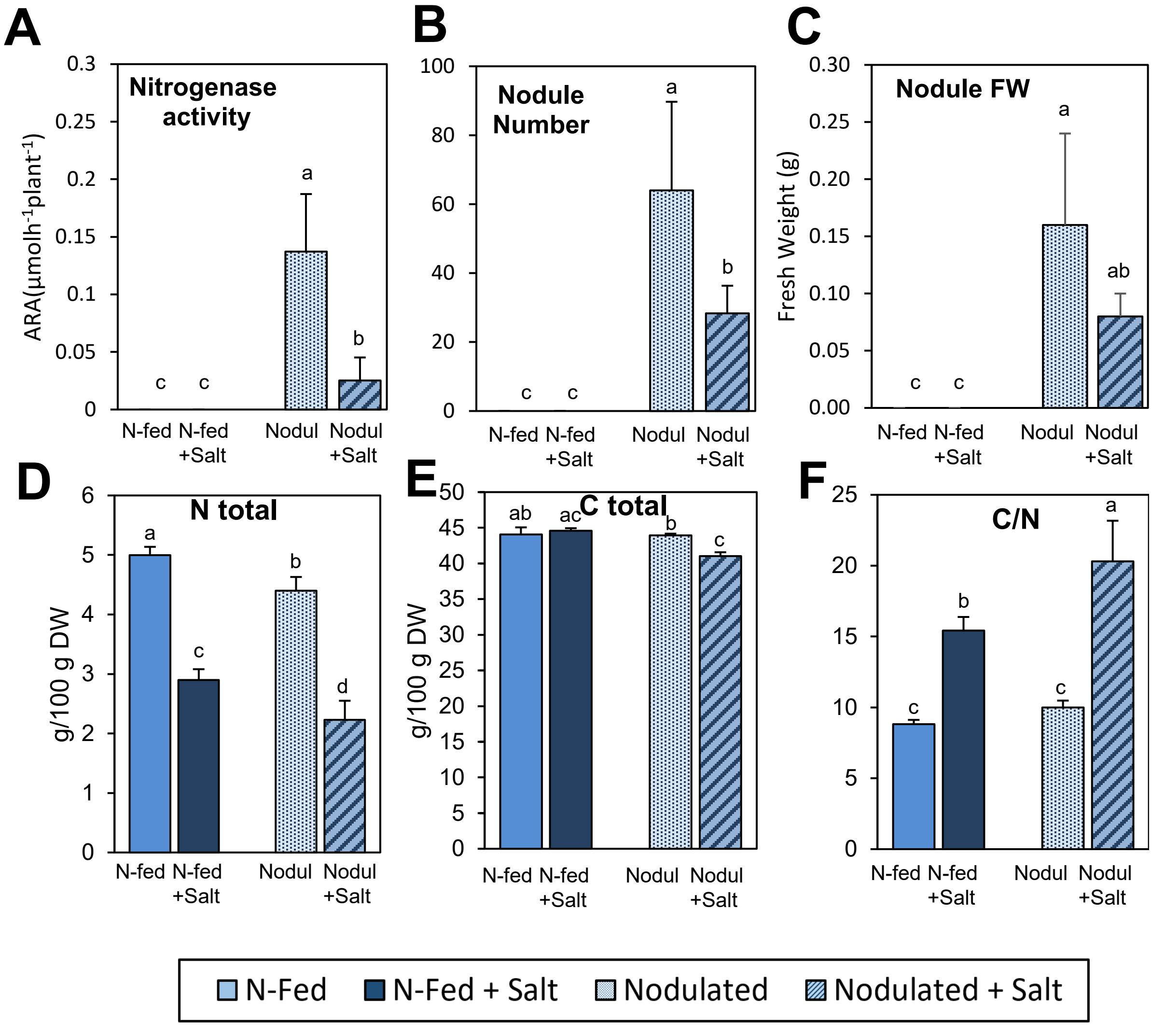

Supplemental Figure S10. Effect of severe salt stress on Rhizobium-vetch symbiosis. A. Nitrogenase activity per plant by Acetylene Reduction Assay (ARA) method**. B.** Nodule number per plant. **C.** Nodule total fresh weight per plant. Quantification of N (**D**) and C (**E**) content and C/N ratios (**F**) on shoot at indicated conditions. All measurements in panels A-F were performed on 49-day-old plants under severe salinity (150 mM NaCl; 21-day treatment applied to 4-week-old plants) or untreated. At least three independent experiments were performed (n=6 plants each). Values are means ± sd. Letters indicate significant differences. *p*<0.01 (ANOVA and Tukey HSD test).

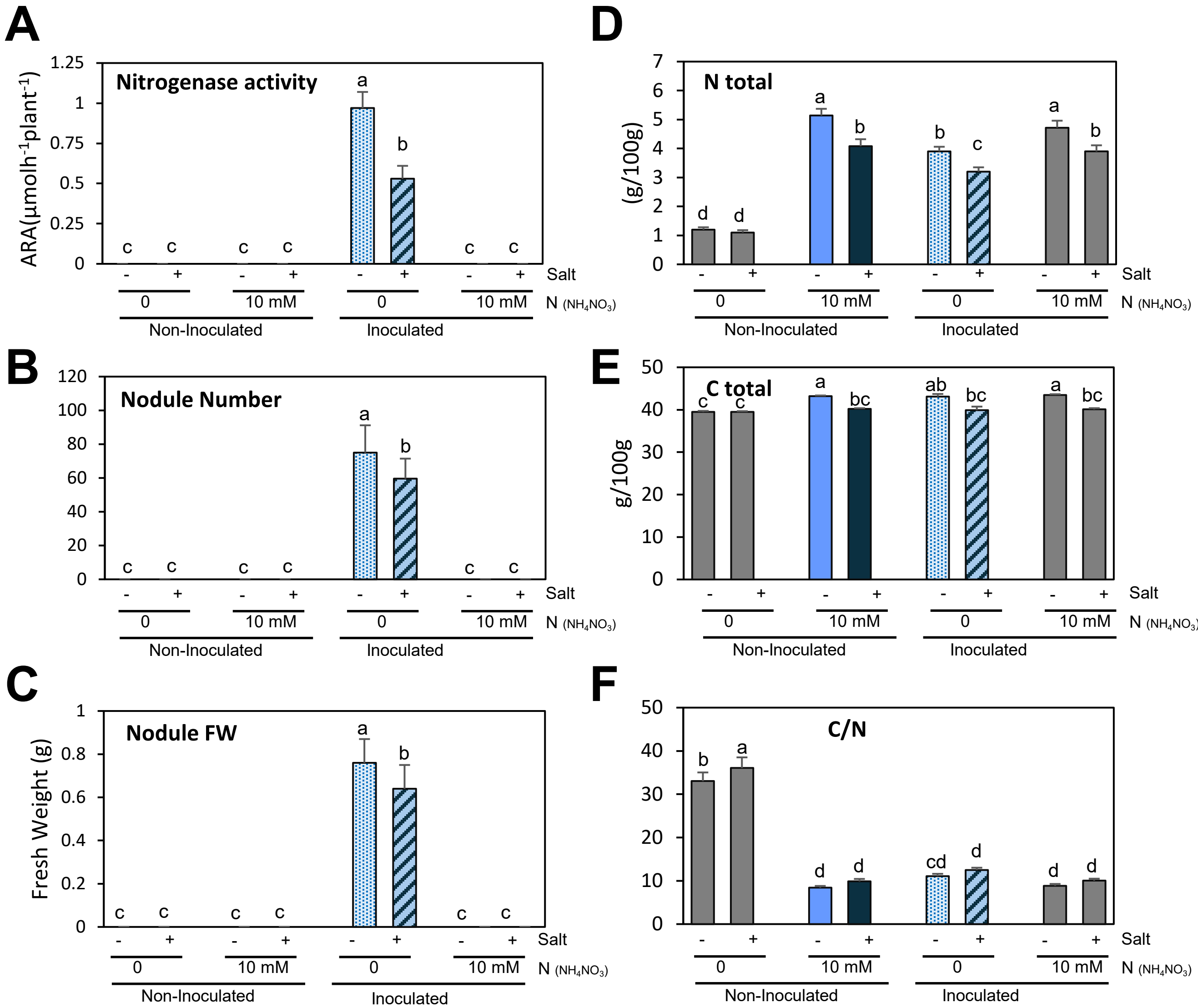

Supplemental Figure S11. Effect of nitrogen concentration on Rhizobium-vetch symbiosis under salt stress conditions. A. Nitrogenase activity per plant by Acetylene Reduction Assay (ARA) method**. B.** Nodule number per plant. **C.** Nodule total fresh weight per plant. Quantification of N (**D**) and C (**E**) content and C/N ratios (**F**) on shoot at indicated conditions. All measurements in panels A-F were performed on 39-day-old plants under severe salinity (150 mM NaCl; 11-day treatment applied to 4-week-old plants) or untreated. At least five plants experiments were measured. Values are means ± sd. Letters indicate significant differences. p<0.01 (ANOVA and Tukey HSD test).
