# Supplementary material for: Rhizobial Symbiosis Enhances Salinity Tolerance in Legumes Through Coordinated Physiological and Molecular Responses: Evidence From Common Vetch and Pea
Source: Plant Cell Environ. 2026 Aug 4;49(11):8912–31. doi: 10.1111/pce.70790 (PMC13646694; doi:10.1111/pce.70790)
Supplement: Supplementary file 2 [file PCE-49-8912-s002.docx]

|  | **Supplemental Table S1:** Summary statistics of *V. sativa* shoot (a) and *root* (b) transcriptome sequencing (Trimming Data Stats) | | | | | | | | | | | | | | | | | |  | |  | |  | |
| --- | --- | --- | --- | --- | --- | --- | --- | --- | --- | --- | --- | --- | --- | --- | --- | --- | --- | --- | --- | --- | --- | --- | --- | --- |
|  | | |  | |  | |  | |  | | |  |  |  | |  | |  | |  | |  | |  |
|  |  | **sample ID** | | **raw_reads** | | **raw_bases** | | **clean_reads** | | **clean_bases** | **error_rate** | | **Q20 (%)** | | **Q30 (%)** | | **GC (%)** | |  | |  | |  | |
|  |  | A01_Ino | | 25 373 151 | | 7.6 | | 25 022 820 | | 7.5 | 0.03 | | 97.6 | | 93.2 | | 41.5 | |  | |  | |  | |
|  |  | A02_Ino | | 23 226 909 | | 7.0 | | 22 827 496 | | 6.9 | 0.03 | | 97.7 | | 93.5 | | 41.9 | |  | |  | |  | |
|  |  | A03_Ino | | 21 237 966 | | 6.4 | | 20 945 669 | | 6.3 | 0.03 | | 97.5 | | 93.1 | | 41.4 | |  | |  | |  | |
|  |  | A04_InoS | | 26 100 797 | | 7.8 | | 25 519 426 | | 7.7 | 0.03 | | 97.8 | | 93.7 | | 42.3 | |  | |  | |  | |
|  |  | A05_InoS | | 31 753 964 | | 9.5 | | 31 111 145 | | 9.3 | 0.03 | | 97.7 | | 93.4 | | 41.6 | |  | |  | |  | |
|  |  | A06_InoS | | 25 388 099 | | 7.6 | | 25 086 392 | | 7.5 | 0.02 | | 98.0 | | 94.2 | | 41.8 | |  | |  | |  | |
|  |  | A07_N | | 28 719 410 | | 8.6 | | 28 035 612 | | 8.4 | 0.03 | | 97.7 | | 93.3 | | 41.9 | |  | |  | |  | |
|  |  | A08_N | | 20 962 072 | | 6.3 | | 20 581 798 | | 6.2 | 0.02 | | 98.1 | | 94.3 | | 42.2 | |  | |  | |  | |
|  |  | A09_N | | 28 540 890 | | 8.6 | | 26 839 802 | | 8.1 | 0.03 | | 97.5 | | 93.1 | | 40.8 | |  | |  | |  | |
|  |  | A10_NS | | 25 859 706 | | 7.8 | | 25 409 356 | | 7.6 | 0.03 | | 97.7 | | 93.5 | | 41.9 | |  | |  | |  | |
|  |  | A11_NS | | 26 906 709 | | 8.1 | | 26 569 117 | | 8.0 | 0.03 | | 97.3 | | 92.8 | | 41.0 | |  | |  | |  | |
|  |  | A12_NS | | 26 369 797 | | 7.9 | | 25 854 586 | | 7.8 | 0.02 | | 98.2 | | 94.5 | | 42.1 | |  | |  | |  | |
|  |  | R13_Ino | | 25 984 029 | | 7.8 | | 25 410 518 | | 7.6 | 0.03 | | 97.8 | | 93.6 | | 41.0 | |  | |  | |  | |
|  |  | R14_Ino | | 23 268 933 | | 7.0 | | 21 763 390 | | 6.5 | 0.03 | | 97.3 | | 92.7 | | 41.4 | |  | |  | |  | |
|  |  | R15_Ino | | 30 868 064 | | 9.3 | | 30 436 537 | | 9.1 | 0.03 | | 97.4 | | 92.9 | | 41.3 | |  | |  | |  | |
|  |  | R16_InoS | | 23 575 655 | | 7.1 | | 23 137 199 | | 6.9 | 0.03 | | 97.8 | | 93.5 | | 41.6 | |  | |  | |  | |
|  |  | R17_InoS | | 30 129 899 | | 9.0 | | 29 569 820 | | 8.9 | 0.03 | | 97.6 | | 93.3 | | 41.4 | |  | |  | |  | |
|  |  | R18_InoS | | 31 263 324 | | 9.4 | | 30 623 572 | | 9.2 | 0.02 | | 98.1 | | 94.4 | | 41.2 | |  | |  | |  | |
|  |  | R19_N | | 33 582 255 | | 10.07 | | 33 253 387 | | 10.0 | 0.03 | | 97.2 | | 92.69 | | 39.9 | |  | |  | |  | |
|  |  | R20_N | | 28 367 216 | | 8.51 | | 27 509 564 | | 8.3 | 0.03 | | 97.14 | | 92.28 | | 42.0 | |  | |  | |  | |
|  |  | R21_N | | 25 840 825 | | 7.75 | | 25 222 986 | | 7.6 | 0.02 | | 98.06 | | 94.19 | | 41.3 | |  | |  | |  | |
|  |  | R22_NS | | 26 902 256 | | 8.07 | | 26 473 571 | | 7.9 | 0.03 | | 97.89 | | 93.94 | | 41.2 | |  | |  | |  | |
|  |  | R23_NS | | 34 631 046 | | 10.39 | | 33 987 234 | | 10.2 | 0.03 | | 97.48 | | 93.17 | | 41.7 | |  | |  | |  | |
|  |  | R24_NS | | 21 870 383 | | 6.56 | | 21 519 064 | | 6.5 | 0.03 | | 97.12 | | 92.46 | | 40.4 | |  | |  | |  | |
|  |  |  | |  | |  | |  | |  |  | |  | |  | |  | |  | |  | |  | |
|  |  | **sample ID:** SampleID R: Root samples; A: Shoot samples | | | | | | | |  |  | |  | |  | |  | |  | |  | |  | |
|  |  | **raw_reads:** Reads count from the raw data, four rows as a unit, with statistics of reads count for every sequencing. | | | | | | | | | | | | | | |  | |  | |  | |  | |
|  |  | **raw_bases:** Base number of raw data. (number of raw reads) * (sequence length), converting unit to G. | | | | | | | | | | | | | | |  | |  | |  | |  | |
|  |  | **clean_reads:** Base number of raw data after filtering. (number of clean reads) * (sequence length), converting unit to G. | | | | | | | | | | | | | | | | |  | |  | |  | |
|  |  | **clean_bases:** (clean base=clean reads*150bp) number multiply read length, saved in G unit. | | | | | | | | | | | | |  | |  | |  | |  | |  | |
|  |  | **error_rate:** Average sequencing error rate, which is calculated by Qphred=-10log10(e). | | | | | | | | | | |  | |  | |  | |  | |  | |  | |
|  |  | **Q20(%):** The percentage of the bases whose Q Phred values is greater than 20. (Number of bases with Q Phred value > 20) / (Number of total bases) *100. | | | | | | | | | | | | | | | | | | | | |  | |
|  |  | **Q30(%):** The percentage of the bases whose Q Phred values is greater than 30. (Number of bases with Q Phred value > 30) / (Number of total bases) *100. | | | | | | | | | | | | | | | | | | | | |  | |
|  |  | **GC(%):** The percentage of G&C base numbers of total bases.(G&C base number) / (Total base number)*100. | | | | | | | | | | | | | | |  | |  | |  | |  | |
|  |  |  | |  | |  | |  | |  |  | |  | |  | |  | |  | |  | |  | |

**Supplemental Table S2:** Statistics of clustered transcripts (unigenes) in *V. sativa de novo* transcriptome sequencing

|  |  | **Transcript** | **Unigene** |  |
| --- | --- | --- | --- | --- |
| ***Length distribution*** | |  |  |  |
|  | *Minimun length* | 301 | 301 |  |
|  | *Mean length* | 1641 | 1311 |  |
|  | *Median length* | 1258 | 818 |  |
|  | *Maximun length* | 17171 | 17171 |  |
|  | *N50* | 2395 | 2097 |  |
|  | *N90* | 780 | 534 |  |
|  | *Total nucleotides (millions)* | 270 | 81 |  |
| ***Transcript Length Interval (number)*** | | |  |  |
|  | *200bp-500bp* | 30190 | 17952 |  |
|  | *500bp-1kbp* | 38083 | 17299 |  |
|  | *1k-2kbp* | 46042 | 13326 |  |
|  | *>2kbp* | 49931 | 12952 |  |
|  | *Total* | 164246 | 61529 |  |
|  |  |  |  |  |
| **Min_length:** the minimum length of Transcript/Unigene | | |  |  |
| **Mean_length:** the average length of Transcript/Unigene | | |  |  |
| **Median_length:** the middle length of Transcript/Unigene | | |  |  |
| **Max_length:** the maximum length of Transcript/Unigene | | |  |  |
| **N50:** spliced transcripts are sorted by length from long to short, and the length of spliced transcripts is N50, which can be used to evaluate the splicing effect. | | | | |
| **N90:** spliced transcripts are sorted by length from long to short, and the length of spliced transcripts is N90, which can be used to evaluate the splicing effect. | | | | |
| **Total_nucleotides:** the total number of nucleotides splicing Transcript/UniGene. -N50/N90: Ranking all transcripts by its length from long to short. Adding the length one by one from the first. When the sum is greater than 50%/90% of the total length, the last added sequence length is N50/N90 | | | | |
| **Length_interval:** Represents different length intervals of Transcript/Unigene | | | | |
| **Number of transcripts:** Indicates the number of Transcript in the corresponding length interval | | | | |
| **Number of Unigenes:** Indicates the number of Unigene in the corresponding length interval | | | | |

**Supplemental Table S3:** Annotation result of various databases for *V. sativa* transcriptomic data at indicated database

| **Database** | **Number of Unigenes** | **Percentage (%)** |
| --- | --- | --- |
| NR | 37818 | 61.5 |
| NT | 35583 | 57.8 |
| KO | 14662 | 23.8 |
| SwissProt | 28975 | 47.1 |
| PFAM | 27679 | 45.0 |
| GO | 27679 | 45.0 |
| KOG | 10811 | 17.6 |
| All Databases | 4479 | 7.3 |
| At least one Database | 48246 | 78.4 |
| Total Unigenes | 61529 | 100.0 |

**Supplemental Table S4.** Gene-specific primers used for quantitative real-time PCR assays

| ***Vicia* Gene name** | **Sequence Forward** | **Sequence Reverse** |  |
| --- | --- | --- | --- |
|  |  |  |  |
| VsGAPDH | AGCTTGTGTATCCCCATTCG | TTGCGAAGAAGGCAACCTAT |  |
| VsUBC | GTCACATCCACACCAACGAC | CAGCGAGAGCAGTAGCAGTG |  |
| VsP5CS2 | AGTGAGGGATGCAAAGACTGA | CCGTGTACTTGCATTGTGGAA |  |
| VsWRKY51 | CAAGGCTCAATTTGACATGC | AGGTGTTCACAACCACGACA |  |
| VsMYB4 | TGGTGGCAACAAAAAGAAGA | CACCAATCTCACCAACCTCA |  |
| VsNAC100 | TAGGTGGTGGACGATGTTGA | CCTGCTTCTGTTTGCGGTAT |  |
| VsDREB1A | TGCTTCCTCCTCTTCTTCCA | CAATACGCCTGCCTCAATTT |  |
| VsERF98 | GCCCTAGACCGCTCTCTTTT | AGGCTGCACGAGCTTATGAT |  |
| VsHAK5 | GACAAGTGGAGCCGAGAGAG | ACCACACCTCTTTCCATTGC |  |
| VsDHN1 | AGGTTACGGTGGAACTGGAA | TTCCAGGATGAACATGATGC |  |
| VsTIP4 | GACCACCCCGACATACAAGT | GGGCTTGCTGGTTTTGTTTA |  |
| VsCYP707 | ATTGCAGCAGCGAAATCAC | AAGTGGGATCCATGCTTGTC |  |
| VsPRX8 | AAGAGATGGTCGCTCTCTCG | GCAAACCCATCTTCACCATC |  |
| VsNRT1 | CGTATTGTCCATGGTTGCAG | GCTTATTGGGACCAGTCACG |  |
|  |  |  |  |
|  |  |  |  |
| **Pea Gene name** | **Sequence Forward** | **Sequence Reverse** | **Gene ID** |
|  |  |  |  |
| PsGAPDH | CTATCACAGCTACTCAGAAGAC | GATTCCTCCTTGATAGCAGC | Psat5g094000 |
| PsDREB | AACAAGCATCCACTCTACCG | CTGGAGTTGGGAATGTGCCG | Psat5g021600 |
| PsDehyd | ACCGGGTATGGAACAACTGGC | ACCACCACCACTAGCACCATGT | Psat1g038600 |
| PsLEA | CAATCTCTCCCCCTGTTTCA | AGTTGGATGATCTGGTATTG | Psat4g026680 |

**Supplemental Table S5.** Complete and detailed GO enrichment results, including specific GO categories, associated pathways, and statistical significance values.

**Supplemental Table S6.** **Total ion composition under the indicated treatments in vetch (A) and pea (B).** Measures were done by analysis of inductively coupled plasma optical emission spectrometry (ICS-OES) spectra observed in each sample. The content of Be, Bi, La, Sb, Se, V was also measured with values under detection level (<0.01mg Kg^-1^).

**Supplemental Table S7. Total metabolite composition (μmol g-1 DW) under the indicated treatments in vetch (A) and pea (B).** Measures were done by analysis of ^1^H-NMR spectra observed in each sample
